# Human Sertoli cells support high levels of Zika virus replication and persistence

**DOI:** 10.1038/s41598-018-23899-x

**Published:** 2018-04-03

**Authors:** Anil Kumar, Juan Jovel, Joaquin Lopez-Orozco, Daniel Limonta, Adriana M. Airo, Shangmei Hou, Iryna Stryapunina, Chad Fibke, Ronald B. Moore, Tom C. Hobman

**Affiliations:** 1 Departments of Cell Biology, University of Alberta, Edmonton, Canada; 2 Departments of Medical Microbiology & Immunology, University of Alberta, Edmonton, Canada; 3 Departments of Medicine, University of Alberta, Edmonton, Canada; 4 Departments of Surgery, University of Alberta, Edmonton, Canada; 5 Li Ka Shing Institute of Virology, Edmonton, Canada; 6 Women & Children’s Health Research Institute, Edmonton, Canada

## Abstract

Zika virus is a teratogenic mosquito-transmitted flavivirus that is associated with birth defects in newborns and Guillain–Barré syndrome in adults. The virus can also be sexually transmitted, but currently, very little is known about the cell types supporting virus replication and persistence in human testes. Using primary cell cultures, we observed that Sertoli but not Leydig cells are highly susceptible to Zika virus infection, a process that is dependent on the TAM family receptor Axl. In cell culture, Sertoli cells could be productively infected with Zika virus for at least 6-weeks. Infection of Sertoli cells resulted in dramatic changes to the transcriptional profile of these cells. The most upregulated mRNA in infected cells was basic fibroblast growth factor (FGF2), a cytokine that was found to enhance Zika virus replication and support viral persistence. Together these findings provide key insights into understanding how Zika virus persists in the male reproductive tract and in turn may aid in developing antiviral therapies or strategies to minimize sexual transmission of this pathogen.

## Introduction

Zika virus (ZIKV) is a major arboviral pathogen responsible for a recent pandemic outbreak in South and Central America^1^. It is well established that ZIKV is teratogenic^2^, capable of crossing the placental barrier and causing microcephaly and other neuropathological manifestations in developing fetuses. The wide-spread prevalence of the mosquito vector (*Aedes spp*.) together with the lack of approved vaccines and therapeutics pose significant challenges to health care systems in endemic areas. As well as vector-based transmission, ZIKV infection can occur through sexual contact^3–5^. This phenomenon has been recapitulated in both mouse^6–8^ and macaque models^9,10^. Because the virus can be detected in human semen several months after infection^11^, even pregnant women in non-endemic areas may be at risk of acquiring ZIKV through sexual transmission of ZIKV from male partners returning from endemic areas. The cell types that support viral persistence in the human male reproductive tract and the molecular mechanisms underlying this process are not well understood.

Several studies using mouse models have reported persistence of ZIKV in testicular tissues up to four weeks^6,7^. In male mice ZIKV infection results in severe testicular inflammation, atrophy and infertility, symptoms that do not appear to occur in human males^6,7,12^. As such, the long-term effects of ZIKV infection on human male fertility are not known.

Sertoli cells play integral roles in male fertility by supporting spermatogenesis and maintenance of spermatogonial stem cells (SSCs) population that eventually give rise to mature sperm^13^. Furthermore, Sertoli cells form the testis-blood barrier, which is critical for protecting the male reproductive tract from pathogens^14^. Leydig cells are another important cell type in testes that produce and secrete the male sex hormone testosterone and are required for development of male reproductive tissue and secondary sexual characteristics^15^.

In the present study, we investigated the susceptibility and permissiveness of primary human Sertoli cells and Leydig cells to ZIKV infection. Whereas Sertoli cells supported high levels of replication of both African and American lineages of ZIKV, Leydig cells were relatively resistant to the virus. ZIKV infection of Sertoli cells was dependent on the TAM (Tyro3, Axl, and Mer) family receptor Axl. We observed a comparatively muted antiviral response following ZIKV infection in Sertoli cells which may also underlie the ability of the virus to persist in Sertoli cell cultures for more than a month and by extension, the male reproductive tract.

RNAseq analysis of acutely infected Sertoli cells revealed dysregulation of over 9,000 mRNA transcripts with ISGs being the main group of genes that was upregulated during both acute and persistent ZIKV infection. In addition, a dramatic increase in production and secretion of fibroblast growth factor 2 (FGF2) was found to occur during ZIKV infection. Studies using FGF2 and blocking antibodies revealed an important role for this cytokine in ZIKV replication and persistence. Moreover, as the amount of FGF2 secreted by Sertoli cells determines the balance between maintenance of spermatogonial stem cells and its differentiation^16^, the dysregulation of FGF2 by ZIKV infection may have implications for male fertility. Finally, we show that ZIKV replication in Sertoli cells can be significantly inhibited by a number of drugs including an FGF receptor antagonist thus indicating potential therapeutic options to limit sexual transmission.

## Results

### Sertoli cells support robust ZIKV replication

**Figure 1:**
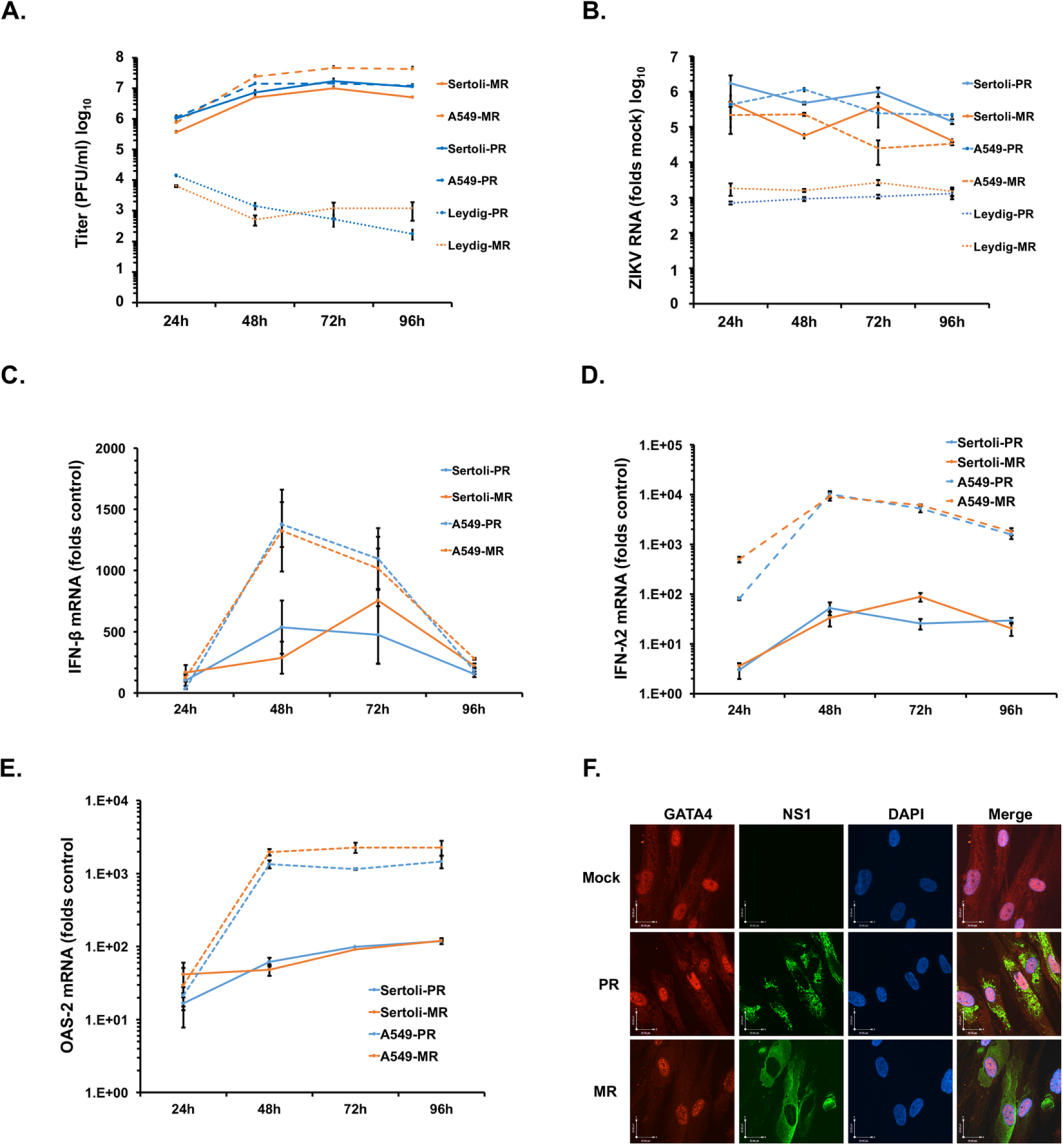
Sertoli cells support high levels of ZIKV replication. (**A**) Primary human Sertoli and Leydig cells and the human A549 cell line were infected with ZIKV strain MR766 (MR) or PRVABC59 (PR) (MOI = 5) for 24, 48, 72 and 96 hours. At each time point, supernatants were harvested and viral titers were determined by plaque assay. (**B**–**E**) Cells were harvested at each time point and viral replication (**B**), Interferon-β (**C**), Interferon-λ2 (**D**) and OAS2 (**E**) mRNA levels were determined by qRT-PCR. (**F**) Sertoli cells seeded on coverslips were infected with ZIKV MR766 (MR) or PRVABC59 (PR) (MOI = 5) for 48 hours and then processed for indirect immunofluorescence using antibodies to ZIKV NS1 and GATA4 to identify infected cells expressing Sertoli markers. Nuclei were stained with DAPI. Representative images are shown. All values are expressed as mean ± standard error. N = 3.

In mouse models, ZIKV infection of the male reproductive tract causes extensive damage to testicular tissue resulting in loss of seminiferous tubule architecture and reduced spermatogenesis^6,7^. As a first step toward understanding how ZIKV infection affects major cell types in human testes, we examined viral replication in human Sertoli cells and Leydig cells. Primary Sertoli cells and Leydig cells as well as A549 cells, a human cell line that is highly susceptible to ZIKV^17^, were infected with African (MR766) and American (Puerto Rican) strains of ZIKV (multiplicity of infection (MOI) = 5) after which viral replication and production were analyzed. Sertoli cells supported high levels of replication for both African and American strains of ZIKV (Figs 1A,B, S1). Peak viral titers reached 10^7^ plaque-forming units (pfu) between three and four days post-infection, which was just slightly lower than those from infected A549 cells. In contrast, replication of ZIKV in Leydig cells was dramatically lower with peak viral titers more than 1000-fold lower than those in Sertoli cells. Because Sertoli cells were much more permissive to ZIKV than Leydig cells, the rest of our studies focused on ZIKV replication in the former cell type.

To determine whether the high susceptibility of Sertoli cells to ZIKV was due to an inability to mount an effective antiviral response, we measured the activation of innate immune genes interferon-β (IFN-B), interferon-λ2 (IFN-λ2), interferon-induced tetratricopeptides-1 (IFIT-1) and oligoadenylate synthase 2 (OAS2) at various times during infection. Compared to A549 cells, induction of innate immune genes especially IFN-λ2 and OAS2 during ZIKV infection was significantly lower in Sertoli cells regardless of whether African or American strains of ZIKV were used for infection (Figs 1C–E, S2). Immunostaining (Fig. 1F) and FACS analysis (Figs S3, S4, Table 1) for ZIKV NS1 protein and the Sertoli cell marker GATA4^18^ revealed that while >90% of the Sertoli cells were positive for GATA4, ~25–45% of the cells were positive for viral antigen 48 h post-infection. The levels of virus-induced apoptosis in Sertoli cells were markedly lower (~4–10%) than in A549 cells (~58–70%) at 72 h post-infection (Fig. S4, Table 1). Of note, the level of apoptosis in Sertoli cells infected with ZIKV PRVABC59 was nearly double that of those infected with the MR766 strain (Fig. S4, Table 1).

**Table 1:** Apoptosis in ZIKV-infected Sertoli and A549 cells.

Sertoli Cells		ZIKV (+)	Casp-3 (+)	Casp-3 (+) in ZIKV (+)
A				
48 h	Mock	1.2 ± 0.06	1.57 ± 0.03	0
	PR	24.3 ± 0.92	1.13 ± 0.09	2.83 ± 0.49
	MR	44.57 ± 0.45	0.93 ± 0.09	1.43 ± 0.18
72 h	Mock	1.27 ± 0.07	1.73 ± 0.26	0
	PR	29.37 ± 1.19	3.97 ± 0.23	9.43 ± 0.52
	MR	43.67 ± 2.66	2.63 ± 0.35	4.43 ± 0.27
B				
*A549 Cells*		**ZIKV (+)**	**Casp-3 (+)**	**Casp-3 (+) in ZIKV (+)**
48 h	Mock	0	0.67 ± 0.03	0
	PR	52.73 ± 2.14	34.0 ± 2.68	41.8 ± 2.98
	MR	79.37 ± 0.99	29.43 ± 1.7	31.17 ± 1.35
72 h	Mock	0	0.83 ± 0.03	0
	PR	48.07 ± 2.94	39.23 ± 2.92	58.57 ± 1.32
	MR	76.87 ± 1.33	59.07 ± 2.05	66.83 ± 1.79

Sertoli cells (A) or A549 cells (B) were infected with ZIKV MR766 (MR) or PRVABC59 (PR) (MOI = 5) for 48 and 72 h. At each time point, cells were harvested and levels of viral infection and apoptosis were determined by FACS. Antibodies against ZIKV NS1 and activated caspase 3 were used to detect infected and apoptotic cells respectively. The percentage of cells positive for ZIKV antigen, activated caspase 3 or both markers are shown. All values are expressed as mean ± standard error. N = 3.

### Axl is an important ZIKV entry receptor in Sertoli cells

**Figure 2:**
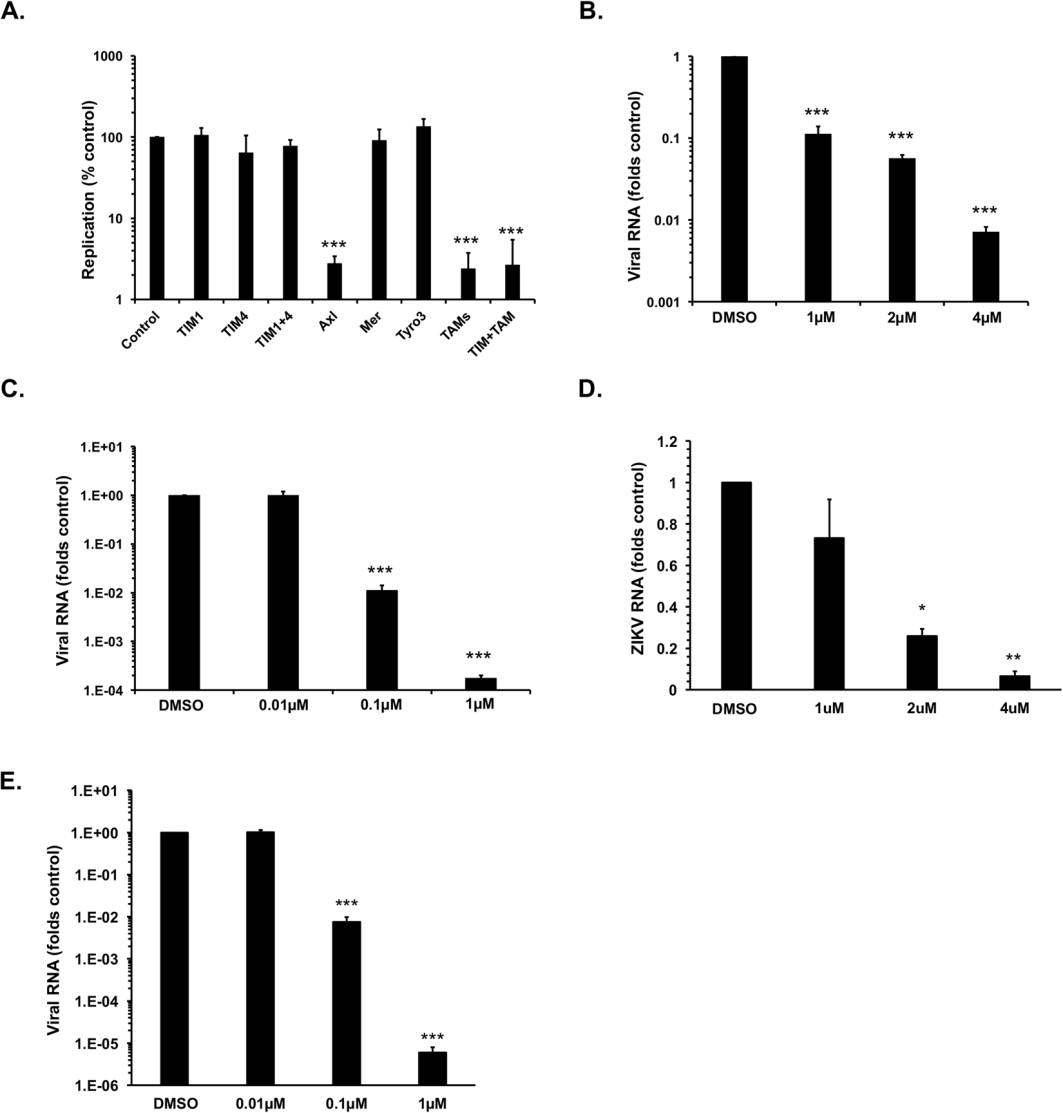
Zika virus entry in Sertoli cells. Sertoli cells were pre-treated with antibodies to the indicated proteins (10 µg/ml) (**A**) or Axl inhibitor (R428) (**B**,**D**) for two hours and then infected with ZIKV PRVABC59 (PR) (**A**,**B**) or MR766 (MR) (**D**) (MOI = 1) for 48 hours after which total RNA was extracted and virus replication determined by qRT-PCR. ZIKV inocula ZIKV PRVABC59 (**C**) or MR766 (MR) (**E**) were incubated with indicated concentrations of duramycin at 37 °C for two hours and then used to infect Sertoli cells (MOI = 1) for 48 hours after which total RNA was extracted and virus replication determined by qRT-PCR. All values are expressed as mean ± standard error. *P < 0.05, **P < 0.01, **P < 0.001 (One-way ANOVA); N = 3.

Several groups of viruses exploit the phosphatidyl serine (PS)- and phosphatidyl ethanolamine (PE)-binding T-cell/transmembrane immunoglobulin and mucin (TIM) and TAM family receptors to enter host cells^19^. For example, ZIKV entry into human astrocytes, skin fibroblasts and endothelial cells is reportedly mediated by these receptors^20–23^. To determine whether ZIKV also exploits these receptors to enter Sertoli cells, blocking antibodies to the TIM receptors (TIM-1 and TIM-4) and TAM receptors (Axl, Tyro-3 and Mer) were added to cells prior to infection with ZIKV. While anti-Axl antibody strongly reduced ZIKV infection, antibodies targeting other receptors (TIM1, TIM4, Mer and Tyro3) did not significantly affect viral replication (Fig. 2A). Approximately 90% of Sertoli cells expressed detectable levels of Axl which were seen to decrease following ZIKV infection (Fig. S6). Interestingly, over 75% of the Leydig cells also expressed Axl (Fig. S7) suggesting that viral entry is not the limiting factor for ZIKV replication in these cells. To further confirm the dependence of ZIKV infection on Axl, we treated Sertoli cells with the Axl-inhibitor R428 and the PS/PE-binding compound duramycin. Both R428 and duramycin significantly inhibited ZIKV replication in a dose-dependent manner (Figs 2B–E, S9E,F) confirming the important role of this receptor for ZIKV infection of Sertoli cells. To determine whether Axl was also important for replication of viral RNA, we pre-treated cells with blocking antibodies to Axl prior to infection with ZIKV. While the number of virus-positive cells was reduced over 75%, the viral antigen signal in virus-positive cells was only reduced marginally indicating Axl is predominantly involved in viral entry (Fig. S5).

### ZIKV persists in Sertoli cell cultures

**Figure 3:**
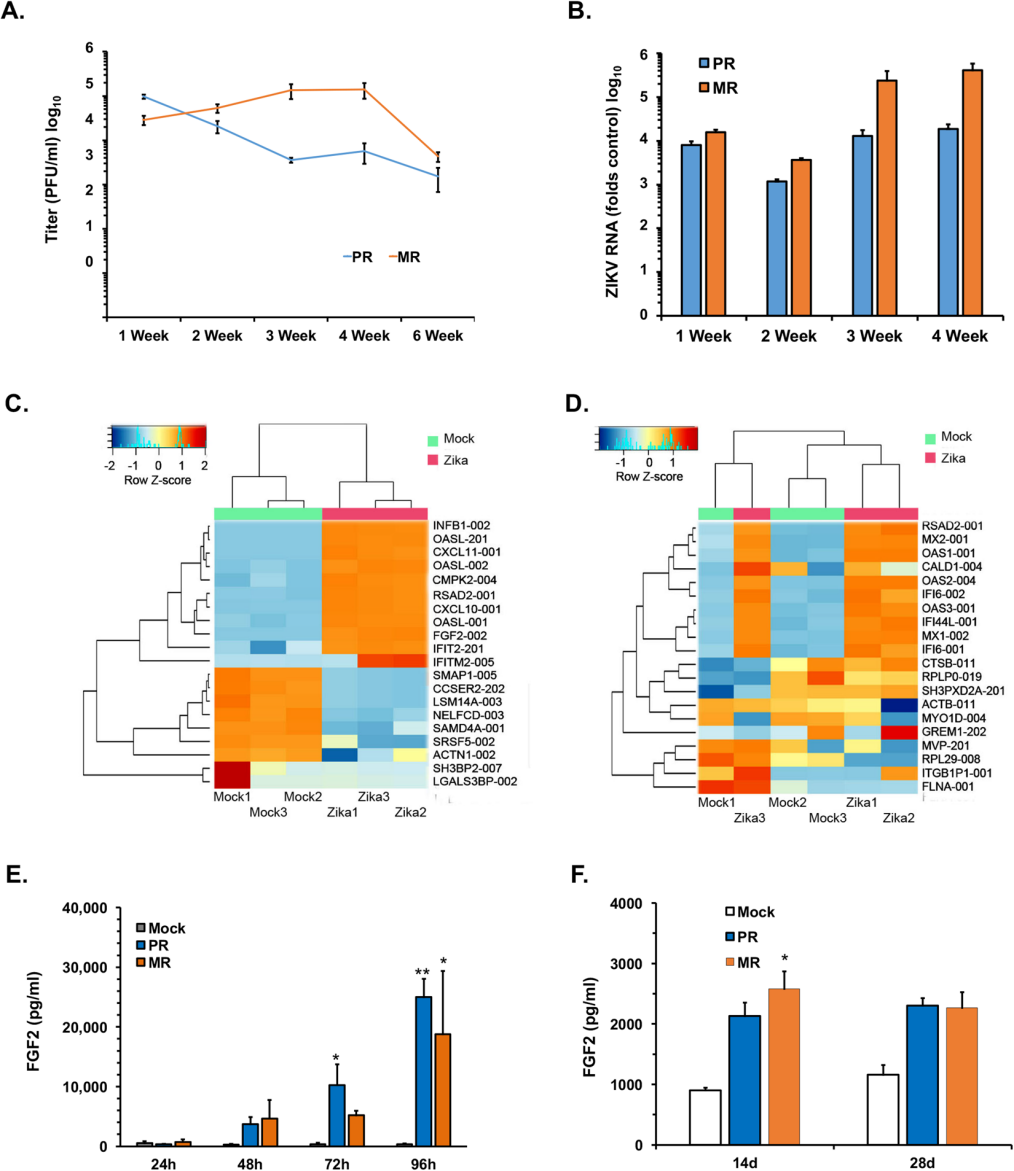
ZIKV persistence and deregulation mRNA transcripts in Sertoli cell. (**A**,**B**) Sertoli cells were infected with ZIKV MR766 (MR) or PRVABC59 (PR) (MOI = 0.5). Samples were harvested at the indicated time points after which viral titers (**A**) and replication (**B**) were determined by plaque assay and qRT-PCR respectively. (**C**,**D**) Sertoli cells were infected PRVABC59 (PR) (MOI = 5 or 0.5 for acute infection or persistent infection respectively) and harvested at 48-hours post-infection or 6-weeks post-infection. The mRNA transcript levels in each sample were determined by RNA-seq analysis. Heat maps representing the 20 transcripts with the largest variance subjected to hierarchical clustering at 48 h post-infection (**C**) and at 6-weeks post-infection (**D**) are shown. (**E**,**F**) Supernatants from Sertoli cells mock-treated or infected with ZIKV MR766 (MR) or PRVABC59 (PR) strains (MOI = 5) were subjected to ELISA to determine levels of FGF2 protein during acute (**E**) and persistent (**F**) infection. All values are expressed as mean ± standard error. *P < 0.05, **P < 0.01 (One-way ANOVA); N = 3.

**Figure 4:**
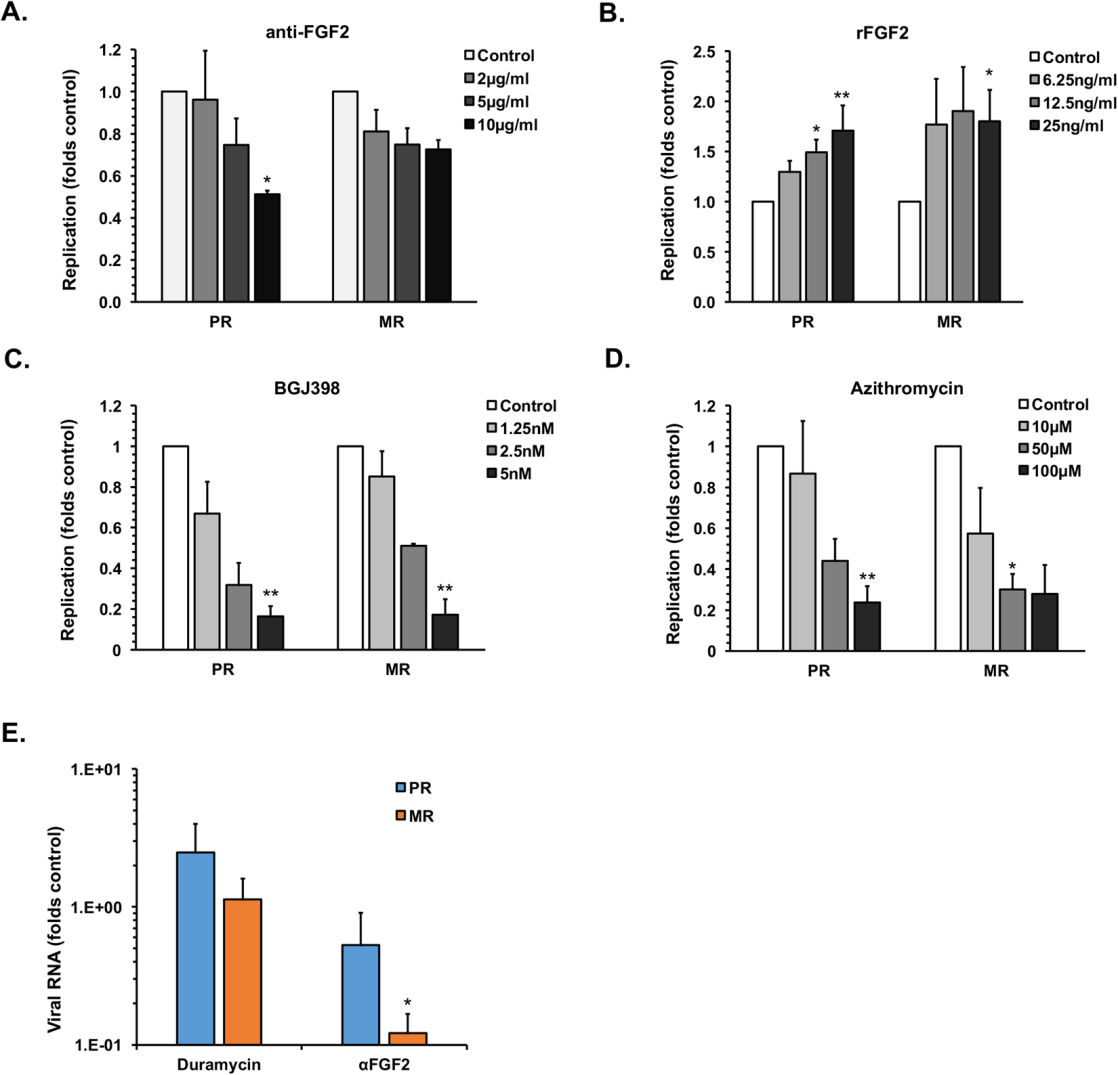
FGF-2 secretion from Sertoli cells supports ZIKV infection and persistence. (**A**–**D**) Sertoli cells were treated for 16-hours with anti-FGF2 antibody (**A**), recombinant human FGF2 (**B**), FGFR inhibitor BGJ398 (**C**) or Azithromycin (**D**) and then infected with ZIKV MR766 (MR) or PRVABC59 (PR) (MOI = 0.5). Cells were harvested 48-hours post-infection and viral replication was determined by qRT-PCR analyses of total RNA extracted from cells. (**E**) Sertoli cells persistently infected with ZIKV MR766 (MR) or PRVABC59 (PR) were treated with DMSO control, duramycin (0.1 uM), control antibody (10 µg/ml) or anti-FGF2 antibody (10 µg/ml) for 7-days and viral replication was determined by qRT-PCR analyses of total RNA extracted from cells. All values are expressed as mean ± standard error. *P < 0.05, **P < 0.01 (One-way ANOVA); N = 3.

In male patients infected with ZIKV, the virus can be detected in semen several months after acute infection^24^. Because Sertoli cells are highly permissive for ZIKV infection, we next asked whether these cells could be persistently infected with ZIKV. Sertoli cells were infected with ZIKV (MOI = 0.5) and then monitored for viral replication in culture for up to 6-weeks. We observed that both African and American strains of ZIKV persisted in Sertoli cells for the entire 6-week period during which time continuous viral replication and shedding observed (Fig. 3A,B). At 6-weeks post-infection, ~15% of the Sertoli cells exhibited detectable ZIKV antigen of which ~90% were GATA4-positive (Fig. S8, Table 2). To determine whether continuous spread was required to maintain viral persistence, persistently infected cultures were treated with the entry inhibitor duramycin for 7-days after which viral RNA was quantitated by qRT-PCR. Duramycin treatment did not significantly affect viral replication indicating that infected Sertoli cells can harbor active virus for extended periods of time (Figs 4E, S9G). Taken together our data are consistent with a scenario in which Sertoli cells are a major viral reservoir for ZIKV in human testes.

**Table 2:** GATA4 expression in ZIKV persistently-infected Sertoli cells.

	ZIKV (+)	GATA4 (+)	GATA4 (+) in ZIKV (+)
PR	14.87 ± 0.52	69.6 ± 6.50	90.77 ± 1.13
MR	18.53 ± 1.52	75.3 ± 8.37	87.03 ± 2.94

Sertoli cells were infected with ZIKV MR766 (MR) or PRVABC59 (PR) (MOI = 0.5). At 42-days post-infection, cells were harvested and the percentage of cells positive for viral antigen and GATA4 expression were determined by FACS. The percentage of cells positive for ZIKV NS1, GATA4 or both markers are shown. All values are expressed as mean ± standard error. N = 3.

### ZIKV infection of Sertoli cells results in major dysregulation of gene expression

Sertoli cells secrete chemokines and cytokines that are essential for maintenance and differentiation of SSCs^25^. As such, virus-induced dysregulation of gene expression may be expected to have significant effects on spermatogenesis and fertility. To assess the effect of ZIKV infection on the gene expression profile of Sertoli cells, we compared transcriptome profiles in mock- and ZIKV-infected (MOI = 5) cells at 48 h post-infection using RNAseq analysis. Resulting data were analyzed using the Kallisto/Sleuth pipeline^26,27^ and a principal component analysis (PCA) was conducted to assess the degree of divergence between transcriptomes in mock- and ZIKV-infected Sertoli cells. The first component of the PCA explained 57% of the variance and clearly separated ZIKV-infected from mock samples. While the variability among ZIKV-infected samples was rather small, mock samples were more variable (Fig. S10A).

At 48 h post-infection, we identified 9,209 transcripts that were differentially expressed (Fig. S10; Table S2). Among these, 5,828 transcripts were upregulated and 3,382 were downregulated. The differential expression of the upregulated transcripts was generally larger than those of downregulated transcripts. Intriguingly, the mRNA transcript that was most highly induced by ZIKV infection encodes fibroblast growth factor 2 (FGF2-002; folds change (FC) = 8,607), a key cytokine secreted by Sertoli cells that has reported roles in spermatogenesis^16,28^. Several interferon-stimulated genes (ISGs) including IFN-B 1 (FC = 1,638), OAS2-like (FC = 1,536), and the C-X-C motif chemokine ligand 11 (FC = 1,242) were also upregulated during acute infection. The only transcript that was downregulated more than 1,000-fold encodes the processing body assembly factor, LSM14A (FC = 1,002), which is also a key viral RNA sensor essential for efficient induction of IFN-β^28^. The identities of the 20 transcripts with the largest variance are shown in Fig. 3C.

Because ZIKV can persist in Sertoli cells for over 6-weeks, we also conducted RNAseq on mock- and ZIKV-chronically infected cells. The degree of virus-induced deregulation of host mRNAs was much less pronounced during chronic infection compared to acute infection (Table S2). The second component of a PCA separated mock from ZIKV persistently infected samples, but accounts for only 23% of the variance (Fig. S10D). This suggests that the transcriptome profile of persistently infected cells was more similar to that of mock cells at 6-weeks post-infection than it was at 48 h post-infection (Fig. S10).

Only 42 transcripts were significantly deregulated at 6-weeks post-infection compared to >9000 at 48 h post-infection. Among those, 40 were upregulated and two were downregulated (Fig. 3D). Thirty-five of the upregulated transcripts were common among the 48 h post-infection and 6-weeks post-infection samples. Expression of the most upregulated transcript was increased almost 800-fold. Not unexpectedly, the top upregulated transcripts were ISGs coding for the MX dynamin-like GTPase-2 (FC = 798), the interferon alpha inducible protein 27 (FC = 250), the UDP-glucose 6 dehydrogenase (FC = 150), the OAS 1 (FC = 137) and the cytidine monophosphate UMP-CMP kinase 2 (FC = 126). Among the downregulated transcripts in cells chronically infected with ZIKV, those encoding splicing factor SWAP homolog (SFSWAP) and the cramped chromatin regulator homolog 1 (CRAMP1) were reduced 82- and 244-fold respectively.

To facilitate interpretation of differential expression analyses results, we conducted gene ontology (GO) analysis^29^ of the top deregulated transcripts (Fig. S11A). For the top 65 upregulated transcripts at 48 h post-infection (FC > 200), the main deregulated terms included positive regulation of endothelial cell proliferation, positive regulation of endoplasmic reticulum stress-induced intrinsic apoptotic signaling pathway and type I interferon signaling pathway. When the GO analysis was extended to all transcripts upregulated at 48 h post-infection, 741 GO terms were found to be significantly overrepresented, which included many related to antiviral defense (Table S3).

For downregulated genes, we first analyzed all transcripts that were deregulated with a FC > 100 (n = 119). The most downregulated terms included regulation in response to oxidative stress, regulation of mRNA splicing via spliceosome, myelination, negative regulation of translation, and terms related to apoptosis regulation (Fig. S11B). GO analysis for all downregulated transcripts yielded 56 terms statistically significantly overrepresented (Table S4).

For the 6-weeks post-infection samples, all 40 upregulated transcripts were subjected to GO analysis. The most overrepresented GO terms were negative regulation of viral genome replication, type I interferon signaling, and interferon gamma signaling pathway. This suggests that the antiviral response is still active to some extent during chronic infection. Since only two transcripts (SFSWAP and CRAMP1) were found downregulated at 6-weeks post-infection, GO analysis did not result in any term overrepresented. Nevertheless, it is expected that down-regulation of SFSWAP may affect the splicing machinery and thus the profile of mRNAs in general^30^. CRAMP1 is poorly characterized but is thought to be involved in chromatin regulation and thus changes in its expression level could also affect transcription.

### FGF2 signaling enhances ZIKV replication in Sertoli cell

The RNAseq analyses indicated that FGF2 mRNA levels were dramatically induced in Sertoli cells during acute ZIKV infection. Next, we used ELISA to determine if there was a corresponding increase in secretion of FGF2 protein. Depending upon the time point and strain of ZIKV used for infection, there was up to 65-fold more FGF2 secreted from ZIKV-infected Sertoli cells (Fig. 3E). As controls, we also measured levels of two other cytokines whose mRNA levels were not significantly affected by ZIKV infection; glial cell line-derived neurotrophic factor (GDNF) and Androgen-binding protein (ABP) following ZIKV infection of Sertoli cells. Consistent with the RNAseq data, we did not observe changes in secretion of these cytokines during viral infection (data not shown). Finally, enhanced FGF2 secretion was also observed in persistently infected samples although the levels were much lower than during acute infection (Fig. 3F).

To investigate the potential role of FGF2 in ZIKV replication, we assessed replication in Sertoli cells that had been pre-treated with a neutralizing antibody to FGF2. Data in Fig. 4A show that virus replication was reduced in a dose-dependent manner (by up to 50%) following anti-FGF2 antibody treatment. The anti-FGF2 antibody had only minimal effects on cell viability (S9A). When FGF2 signaling was blocked by treatment of cells with the FGF receptor inhibitor BGJ398 ZIKV replication was reduced by up to 80% without significant reduction in cell viability (Figs 4C, S9C). These data suggest that FGF2-dependent signal has a proviral effect. Consistent with this conclusion, treatment of Sertoli cells with recombinant FGF2 resulted in a modest but significant increase in ZIKV replication (Figs 4B, S9B). Similarly, treatment of persistently infected Sertoli cells with anti-FGF2 antibody reduced viral replication by 50–80% (Figs 4E, S9G). While upregulation of FGF2 appears to favor virus replication, the mechanism by which this occurs and whether it affects SSC maintenance and differentiation requires further investigation.

Next, we investigated whether the recently reported ZIKV-inhibitor azithromycin^21^ could reduce viral replication in Sertoli cells. Following treatment of cells with azithromycin, viral replication was reduced by over 75% in ZIKV-infected cells (Fig. 4D) with limited effect on cell viability (Fig. S9D). As azithromycin had similar efficacy as abrogation of FGF signaling in blocking viral replication, both azithromycin and FGFR inhibitors may be explored as potential therapeutic options against ZIKV infection. It should be noted that the FGFR inhibitor BGJ398, which is in clinical trials for cancer indications, is >40,000-fold more potent that azithromycin in inhibiting ZIKV replication. Finally, as viral NS1 is a reliable marker for infection by other flaviviruses, we examined whether NS1 is secreted from ZIKV-infected Sertoli cells in detectable quantities. We observed robust secretion of NS1 from ZIKV-infected Sertoli cells with peak levels reaching ~ 350 ng/ml between 48–72 h post-infection (Fig. S13). Our data suggest that secreted NS1 can be used as potential immunodiagnostics marker for ZIKV infection in semen.

## Discussion

ZIKV is unique among mosquito-transmitted flaviviruses in its ability to spread through sexual transmission^3–5^. Although this mode of infection not the major route of transmission in endemic areas, infected males who return from endemic regions pose a significant risk to their partners who reside outside areas where ZIKV is circulating. Several studies have reported the presence of infectious ZIKV in semen several months after exposure to the virus^3–5^. High-level replication of ZIKV in the testes of several animal models including mouse^6–8,12,31^, macaque^9,10^ and hamster^32^ have also been observed. However, the severe inflammation, disruption of tissue architecture and atrophy observed in mouse models^6–8,31^ has not been documented in humans suggesting that there may be significant differences between ZIKV infection of male reproductive tissues in humans and mice.

The present study focused on the susceptibility and permissiveness of two major cell types (Sertoli cells and Leydig cells) supporting spermatogenesis in human testes. The observation that Sertoli cells support high levels of virus replication is in agreement with recent studies in mouse models^6–8^ and human cells^33^. The high susceptibility of these cells to ZIKV is likely due in part to a muted antiviral response. As Sertoli cells maintain the blood-testis barrier, direct infection of these cells may facilitate entry of ZIKV into the lumen of seminiferous tubules allowing infection of developing spermatogonia. Interestingly, in contrast to observations in mouse models^7,12^, human Leydig cells poorly supported virus infection in our hands. Of potential significance for persistence in the male reproductive tract, we observed that Sertoli cells support high levels of virus replication and shedding for prolonged periods of time. Similar to what has been reported in cells from other tissues^20–23^, ZIKV infection of Sertoli cells was dependent on the cell surface receptor Axl, suggesting that this membrane protein is a ubiquitous entry factor for multiple human cell types. In contrast, though Axl is highly expressed in mouse Sertoli cells^7^, it was not essential for viral entry in these cells^6^ thus indicating another key difference between mouse and human Sertoli cells. Interestingly, although Leydig cells express Axl, they do not support efficient ZIKV replication suggesting that the restriction point is further downstream of receptor binding.

RNASeq analysis revealed that acute infection of Sertoli cells with ZIKV results in massive dysregulation of host transcripts (>9,000). The most upregulated transcript encodes the cytokine FGF2, which together with GDNF plays an important role in SSC maintenance and spermatogenesis. Our experiments revealed a pro-viral role for FGF2 in ZIKV replication and persistence. Depending upon the cell type, this growth factor can activate a variety of downstream signaling pathways, some of which can benefit virus replication. For example, FGF2 inhibits apoptosis in several cell types^34,35^ and therefore one obvious benefit of increased FGF2 expression is a bigger window of time for virus replication. The most downregulated transcript in ZIKV infected Sertoli cells was LSM14A, a host-restriction factor required for viral RNA sensing and interferon induction. Its suppression could explain in part the muted interferon response observed in Sertoli cells following ZIKV infection.

To the best of our knowledge, this is the first RNAseq data using ZIKV-infected Sertoli cells. RNAseq and developmental processes are largely tissue-specific, which prevents direct comparison of our data with earlier studies. Previous reports on the transcriptional response induced by ZIKV have focused on neural progenitor cells differentiated from pluripotent stem cells^36^, on stem cell-derived organoids and neurospheres^37^, and in microglia, fibroblast, embryonic kidney and monocyte-derived macrophage cell lines^38^. The unifying theme among these studies is that ZIKV infection induces a robust antiviral response that includes upregulation of ISGs and chemokines. Downregulation of transcripts that are linked to developmental abnormalities is, as expected, different in each tissue/cell type analyzed. For instance, Tang *et al*.^36^ found that in differentiated neuron cells, cell cycle related transcripts were severely downregulated by ZIKV, a situation that could affect development of brain cells. Similarly, Dang *et al*.^37^ observed that genes required for nervous system development and regulation of synapse structure/activity were downregulated during ZIKV infection. In cell lines, gene ontology analysis revealed that ZIKV downregulated genes that affect a variety of metabolic processes^38^. In Sertoli cells, we observed downregulation of mRNAs that encode proteins that function in response to oxidative stress, regulation of mRNA splicing via spliceosome, myelination, negative regulation of translation, and apoptosis, which are consistent with subversion of the immune-system by ZIKV.

Because FGFR signaling inhibitors and azithromycin severely hampered viral replication, it may be worth further exploring their utility as therapeutic options to block sexual transmission of ZIKV by limiting persistence in the male reproductive tract. Finally, we show that infected Sertoli cells robustly secrete NS1 protein which could potentially be exploited as an immunodiagnostic marker for rapid screening of semen from suspected cases of ZIKV infection in male patients.

Together, our data suggest that the high susceptibility of Sertoli cells combined with their ability to sustain ZIKV replication for prolonged periods makes them an ideal candidate for the viral reservoir in testes. In addition, as well as being benefiting virus replication, the observed dysregulation of FGF2 during ZIKV infection may have short and/or long-term implications for fertility.

## Materials and Methods

### Cell culture and virus infection

Primary human Sertoli cells were purchased from Lonza Walkersville, Inc. USA. and cultured in SeBM™ Sertoli Cell Basal Medium (Lonza) supplemented with 100 U/ml penicillin and streptomycin, 5% heat-inactivated fetal bovine serum (FBS; Gibco) at 37 °C in 5% CO_2_. All experiments were carried out with Sertoli cells between passages 3–5. Primary human Leydig cells were purchased from ScienCell Research Laboratories, USA. and grown in Leydig Cell Medium (ScienCell Research Laboratories, USA). All experiments were carried out with Leydig cells after a single passage in culture. A549 cells and Vero cells from the American Type Culture Collection (Manassas, VA) were cultured in Dulbecco’s modified Eagle’s medium (DMEM; Gibco) supplemented with 100 U/ml penicillin and streptomycin, 2 mM glutamine (Gibco), 10% heat-inactivated fetal bovine serum (FBS; Gibco) at 37 °C in 5% CO_2_. The *Aedes albopictus* derived c6/36 cells were kindly provided by Dr. Sonja Best, NIH Rocky Mountain laboratories, Hamilton, Montana, USA and was cultured in Minimal Essential Medium (MEM; Gibco) supplemented with 100 U/ml penicillin and streptomycin, 2 mM glutamine (Gibco), 10% heat-inactivated fetal bovine serum (FBS; Gibco) and 1x non-essential amino acids (Gibco) at 32 °C in 5% CO_2_. The Zika virus (strain PRVABC59) was kindly provided by Dr. David Safronetz at the Public Health Agency of Canada. The Zika virus (strain MR766) was generated from a molecular clone of the virus kindly provided by Dr. Matthew J. Evans at the Icahn School of Medicine at Mount Sinai, New York, USA. All virus manipulations were performed according to level-2 containment procedures. Virus stocks were generated in C6/36 cells and titrated (by plaque assay) using Vero cells.

### Antibodies and reagents

The antibodies were purchased from the following sources: Rabbit anti-GATA4 antibody (Abcam, ab84593), Goat anti-TIM1 antibody (R&D systems, AF1750), Goat anti-TIM4 antibody (R&D systems, AF2929), Goat anti-Tyro3 antibody (R&D systems, AF859), Goat anti-Axl antibody (R&D systems, AF154), Goat anti-Mer antibody (R&D systems, AF891), Mouse anti-FGF2 (EMD Millipore, 05-117), Mouse IgG1 isotype control antibody (R&D systems, MAB002), rabbit anti-active caspase-3 (Cell Signaling, #9664), mouse anti-β-actin (a3853) from Sigma Aldrich. A mouse monoclonal antibody to ZIKV NS1 protein was developed in this laboratory. The reagents were purchased from the following sources: Azithromycin (Sigma Aldrich, PZ0007), Duramycin (Sigma Aldrich, D3168), R428 (Selleckchem, S2841), human bFGF (Sigma Aldrich, F0291) and BGJ398 (Adooq Bioscience, A11159).

### Confocal microscopy

A549 cells and Sertoli cells on coverslips were fixed for 15 min at room temperature with freshly prepared 4% paraformaldehyde (Electron Microscope Sciences) in PBS. Samples were then washed three times with PBS, permeabilized with 0.5% Triton X 100 in PBS for 5 minutes at room temperature, washed three times with PBS and incubated in blocking buffer (5% bovine serum albumin [BSA; Sigma Aldrich] in PBS) at room temperature for 1 h. Incubations with primary antibodies in blocking buffer were carried out at room temperature for 1 h, followed by three washes in PBS. Samples were then incubated with corresponding secondary antibodies in blocking buffer for 1 h at room temperature, followed by three washes in PBS. The secondary antibodies (Invitrogen) were used at 1:1000 dilutions in blocking buffer. Prior to mounting, samples were incubated with DAPI (4′,6-diamidino-2-phenylindole; Sigma Aldrich) (1 μg/ml) for 5 min at room temperature before washing. Coverslips were mounted on microscope slides using Prolong Gold anti-fade mounting reagent (Life Technologies). Images were acquired using an Olympus IX-81 spinning-disk confocal microscope equipped with a 40x/1.42-numerical-aperture oil PlanApo N objective. Images were analyzed using Volocity 6.2.1 software (PerkinElmer).

### Persistence assay

Sertoli cells (passage 3) seeded in 6-well plates were infected with ZIKV MR766 (MR) or PRVABC59 (PR) at MOI of 0.5. The cell culture medium was exchanged twice a week until the cells were harvested at indicated time points. At indicated time points samples were harvested 4-days post medium-change. The cell supernatants were collected, viral titer was determined by plaque assay, and FGF2 levels were determined by ELISA. The cell lysates were harvested and viral replication was determined by qRT-PCR.

### Quantitative real-time PCR (qRT-PCR)

Total RNA from A549, Leydig and Sertoli cells was isolated using the RNA NucleoSpin Kit (Machery Nagel) and reverse transcribed using random primers (Invitrogen) and Improm-II reverse transcriptase (Promega) at 42 °C for 1.5 h. The resulting cDNAs were mixed with the appropriate primers (Integrated DNA Technologies) and the PerfecTa SYBR green SuperMix with Low ROX (Quanta Biosciences) and amplified for 40 cycles (30 s at 94 °C, 40 s at 55 °C and 20 s at 68 °C) in a Stratagene Mx3005P qRT-PCR machine. The gene targets and primers used are listed in Table S8. The ΔCT values were calculated using *β-actin (ACTB)* mRNA as the internal control. The ΔΔCT values were determined using control samples as the reference value. Relative levels of mRNAs were calculated using the formulas 2^(−ΔΔCT)^.

### Flow cytometry

After the indicated experimental treatments, cells were detached from plates with Versene solution (ThermoFisher), pelleted by centrifugation at 1000 g, washed once in PBS, fixed with 2% paraformaldehyde (in PBS) and then permeabilized with PBS containing 0.1% Triton X100. Following incubation with primary and secondary antibodies, samples were analyzed using a Becton-Dickinson LSRFortessa cell analyzer and FACSDiva Software (BD Biosciences). Background fluorescence for viral antigens was set using mock-infected cells while for cellular antigens with cells treated with secondary antibody alone. Cell doublets and debris were excluded from the analyses using forward-scatter-width-discrimination.

### FGF2 ELISA

Cell culture supernatants from mock or Zika-infected samples were collected at various time points and stored at −80 °C. The ELISA was carried out using human bFGF ELISA kit (RayBiotech, ELH-bFGF-1) according to manufacturer’s instructions. Detailed protocol is available on manufacturer’s website. All samples were measured in duplicate and the FGF2 levels were determined using a standard curve generated against known quantities of FGF2.

### NS1 ELISA

Cell culture supernatants from mock or Zika-infected samples were collected at various time points and stored at −80 °C. The ELISA was carried out using an HRP-conjugated anti-ZIKV NS1 monoclonal antibody developed in-house. The NS1 levels were determined from a standard curve generated against known quantities of NS1 (Native Antigen, ZIKVSU-NS1).

### RNAseq libraries

RNAseq libraries were constructed using the TruSeq RNA Sample Prep V2 Kit (Illumina) as per manufacturer instructions. In brief, 1 μg of total RNA was diluted in 50 μL of nuclease-free H_2_O and mixed with one volume of messenger RNA purification beads containing oligos dT conjugated to paramagnetic beads and the suspension was heated to 65 °C for 5 min, cooled down to 4 °C and then incubated at room temperature for 5 min. The suspension was then incubated on a magnetic stand for 5 min at room temperature, the supernatant removed, and the beads washed with 200 μL of beads washing solution. Finally, the RNA was eluted from the beads in 50 μL of elution buffer at 85 °C, for 2 min and then cooled down to 25 °C. RNA was re-bound to magnetic beads to increase specificity adding 50 μL of Bead Binding Buffer and beads were washed as described above. On-beads RNA was supplemented with 19.5 μL Elute, Prime Fragment mix containing random hexamers for cDNA synthesis. The solution was heated at 94 °C for 8 min and then cooled down to 4 °C to allow for annealing of random hexamers. cDNA synthesis was conducted in 8 μL First Strand Master Mix containing SuperScript II reverse transcriptase at a 1:9 ratio using the following thermocycling program 25 °C for 10 min, 42 °C for 50 min, 70 °C for 15 min, hold at 4 °C. Second strand cDNA synthesis was conducted in the presence of 25 μL of Second Strand Master Mix at 16 °C for 1 hr. cDNA reaction was supplemented with 90 μL of AMPure XP beads, incubated for 15 min at room temperature, and then incubated for 5 min on a magnetic stand. Beads were washed twice with 200 μL of 80% ethanol, air-dried, and finally the cDNA was eluted in 50 μL Resuspension Buffer. End repair was conducted in 40 μL of End Repair Mix for 30 min at 30 °C. End-repaired cDNA was supplemented with 160 μL of AMPure XP beads, which were washed and end-repaired cDNA eluted in 15 μL as described above. End-repaired cDNA was 3′ adenylated in 12.5 μL of A-tailing mix with the following thermo cycler program: 37 °C for 30 min, 70 °C for 5 min, 4 °C hold). Adapters were ligated by adding 2.5 μL of Ligation Mix, incubated at 30 °C for 10 min; reaction was stopped with 5 μL of Stop Ligation Buffer. Reaction was cleaned up twice, in the first round with 42 μL of AMPure XP Beads and eluted in 50 μL of Resuspension Buffer, and in the second round with 50 μL AMPure XP Beads and eluted in 20 μL, all as described above. cDNA was enriched by adding 25 μL of PCR Master Mix using the following PCR program: 98 °C for 30 sec; [15X] 98 °C for 10 sec, 60 °C for 30 sec, 72 °C for 30 sec; 72 °C for 5 min; 4 °C hold. PCR reaction was cleaned up with 50 μL of AMPure XP Beads and recovered in 30 μL of Resuspension Buffer, as described above. Libraries were sequenced in a NextSeq instrument, using 75 cycles paired end V3 sequencing kit.

### Bioinformatics

Libraries were de-multiplexed using the appropriate workflow in a NextSeq instrument. After quality control, libraries were pseudo-aligned to the human cDNA database Homosapiens_GRCh38.rel79 with Kallisto^26^. Data was parsed with in-house Python scripts. Differential expression analysis was conducted with the programming language R, using the package Sleuth^27^. Plots were generated with R scripts.

### Statistical analyses

A one-way ANOVA was used for comparison of multiple samples, while paired Student’s *t*-test was performed for pair-wise statistical comparison. The mean ± standard error of the mean is shown in all bar and line graphs. All statistical analyses were performed using GraphPad Prism software.

### Data availability

Sequences of libraries described in this paper are publicly available at the Sequence Read Archive (SRA) portal of NCBI under the accession numbers SRP099470.

## Electronic supplementary material

Supplementary figure legends and table legends

Supplementary Info

Supplementary tables S1-S5
