# Supplementary material for: Human Sertoli cells support high levels of Zika virus replication and persistence
Source: Sci Rep. 2018 Apr 3;8:5477. doi: 10.1038/s41598-018-23899-x (PMC5883016; doi:10.1038/s41598-018-23899-x)
Supplement: Supplementary file 2 — Supplementary Info [file 41598_2018_23899_MOESM2_ESM.pdf]

Title: Human Sertoli cells support high levels of Zika virus replication and persistence

Anil Kumar[1], Juan Jovel[3], Joaquin Lopez-Orozco[1], Daniel Limonta[1], Adriana M. Airo[2], Shangmei Hou[1], Iryna Stryapunina[1], Chad Fibke[2], Ronald B. Moore[4] and Tom C. Hobman*[1,2,5,6]

Supplementary Figures S1-S12

A. **B.**

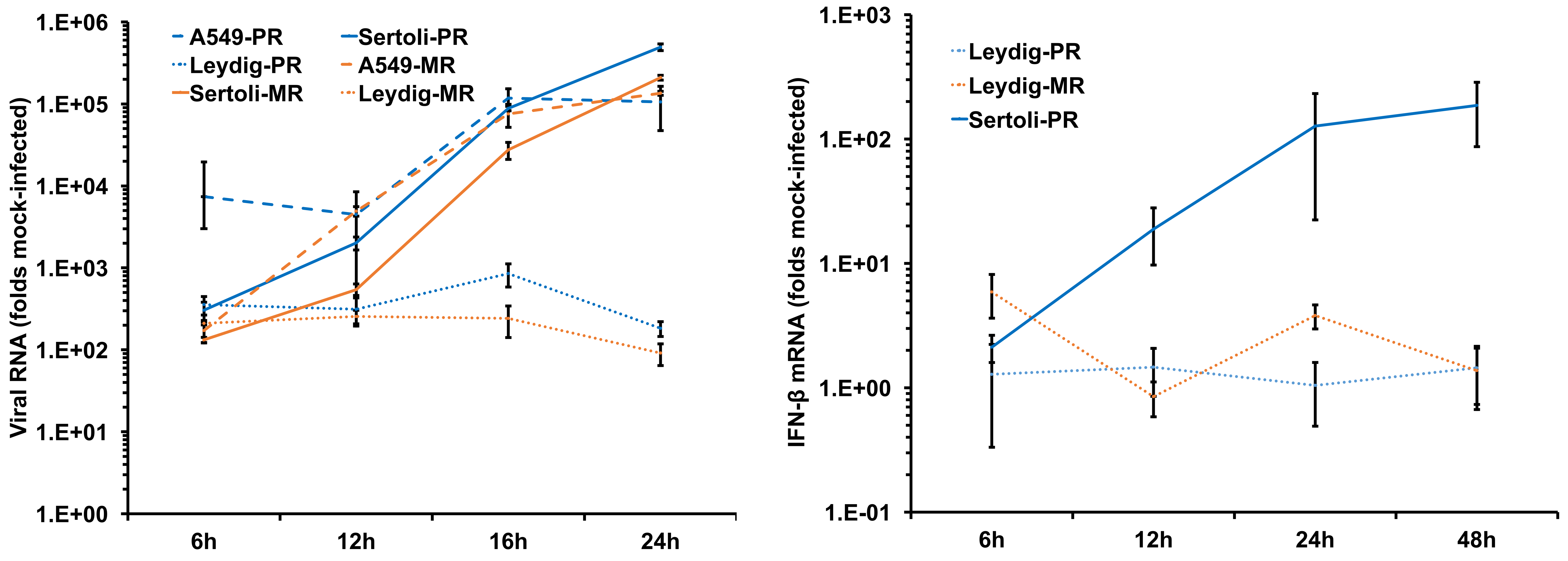

Figure S1. ZIKV replication kinetics at early time points and IFN-β induction in Leydig cells. A. A549 cells, Sertoli cells and Leydig cells were infected with ZIKV MR766 (MR) or PRVABC59 (PR) (MOI=5) for 6, 12, 18 and 24 hours. The cells were harvested at indicated time points and the levels of ZIKV RNA were measured by q-RT PCR. **B.** Sertoli cells were infected with ZIKV PRVABC59 (PR) (MOI=5) while Leydig cells were infected with ZIKV MR766 (MR) or PRVABC59 (PR) (MOI=5) for 6, 12, 24 and 48 h. The cells were harvested at each time point and the levels of ZIKV RNA (A) and IFN-b (B) were measured by q-RT PCR. All values are expressed as mean ± standard error. N=3

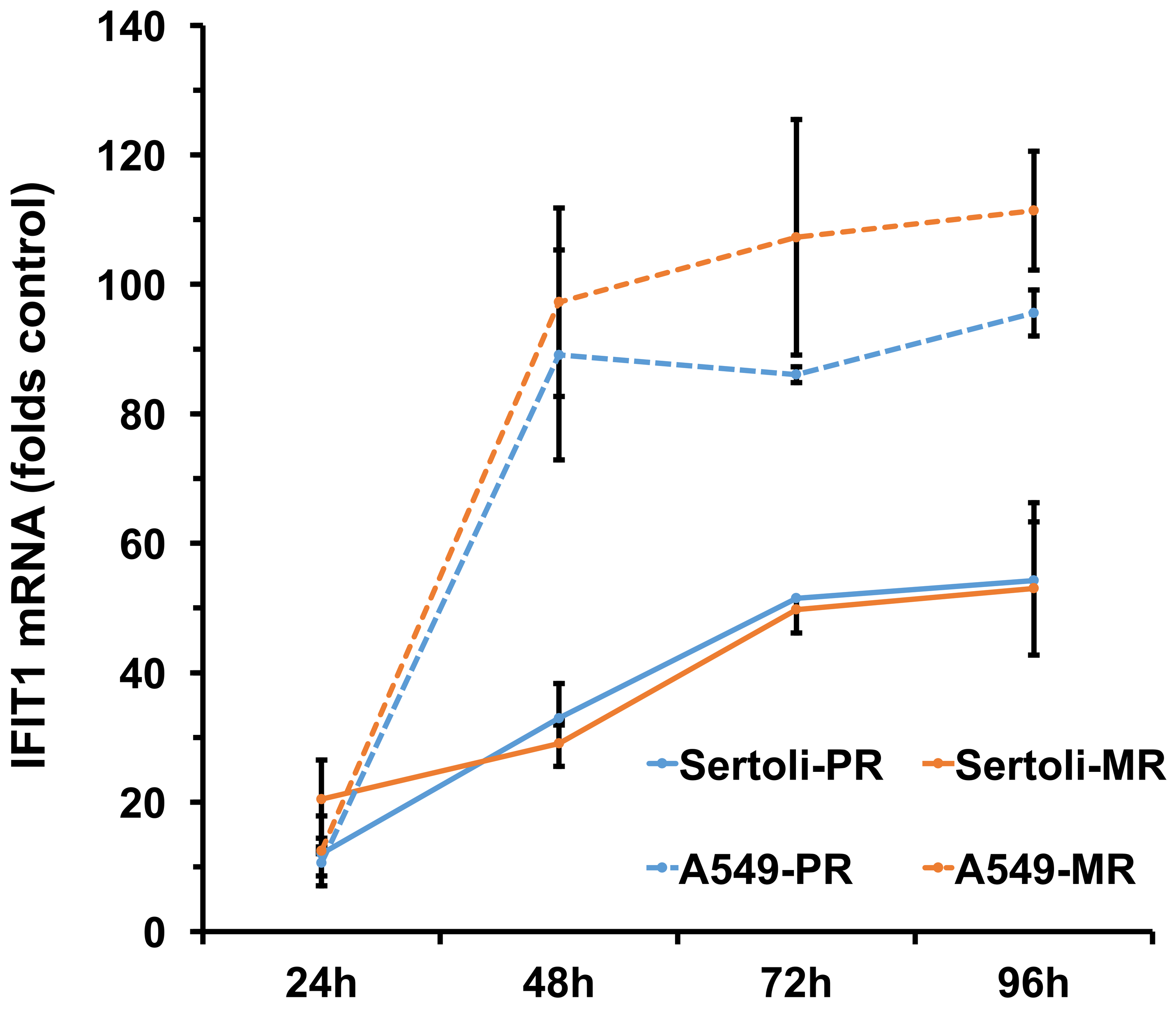

Figure S2. ZIKV infection of Sertoli cells does not robustly induce ISGs. Sertoli cells and A549 cells were infected with ZIKV MR766 (MR) or PRVABC59 (PR) (MOI=5) for 24, 48, 72 and 96 hours. The cells were harvested at each time point and the levels of IFIT1 mRNA were measured by q-RT-PCR. All values are expressed as mean ± standard error. N=3

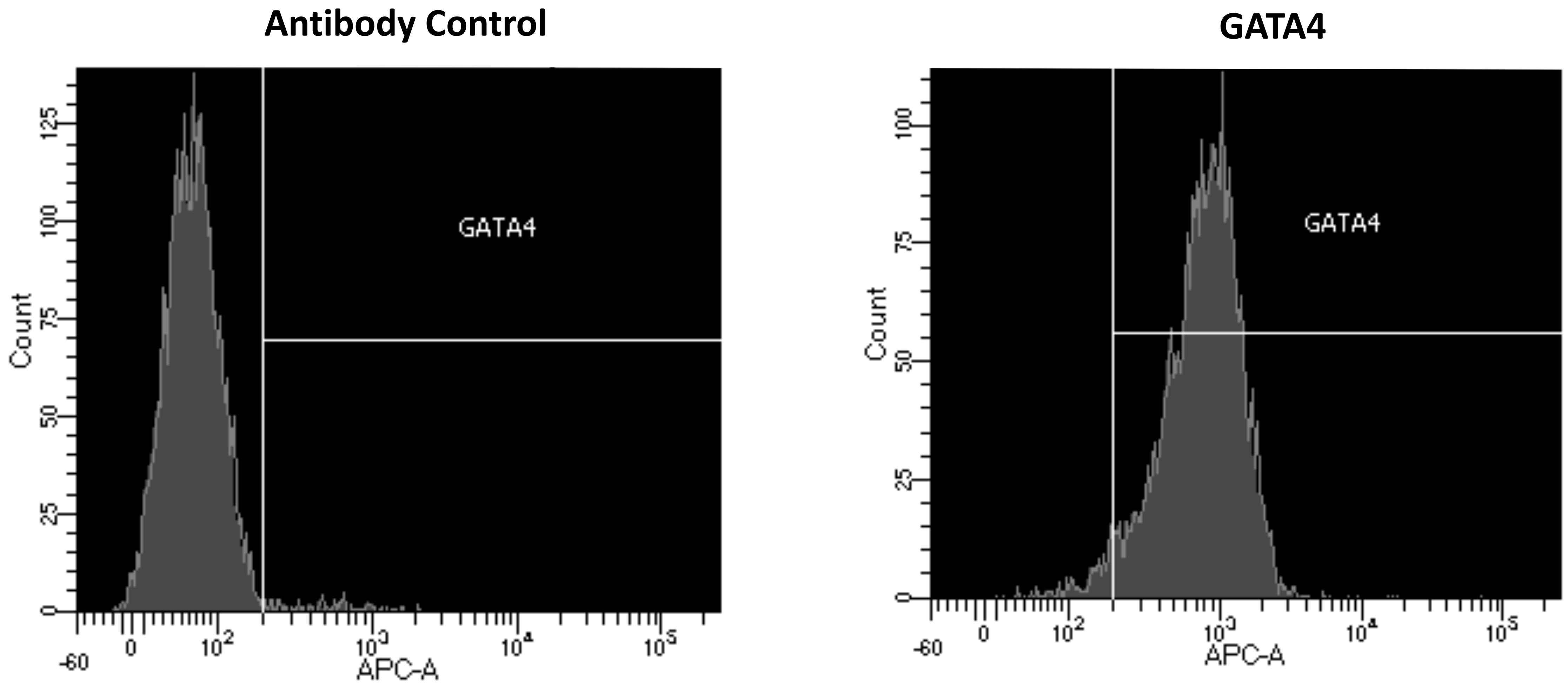

Figure S3. GATA4 expression in Sertoli cell cultures. GATA4 expression within Sertoli cell cultures was determined by FACS after staining with a rabbit anti-GATA4 antibody. The percentage of GATA(+) cells was 93.63 ± 2.89. Representative histogram images are shown. N=3.

A.

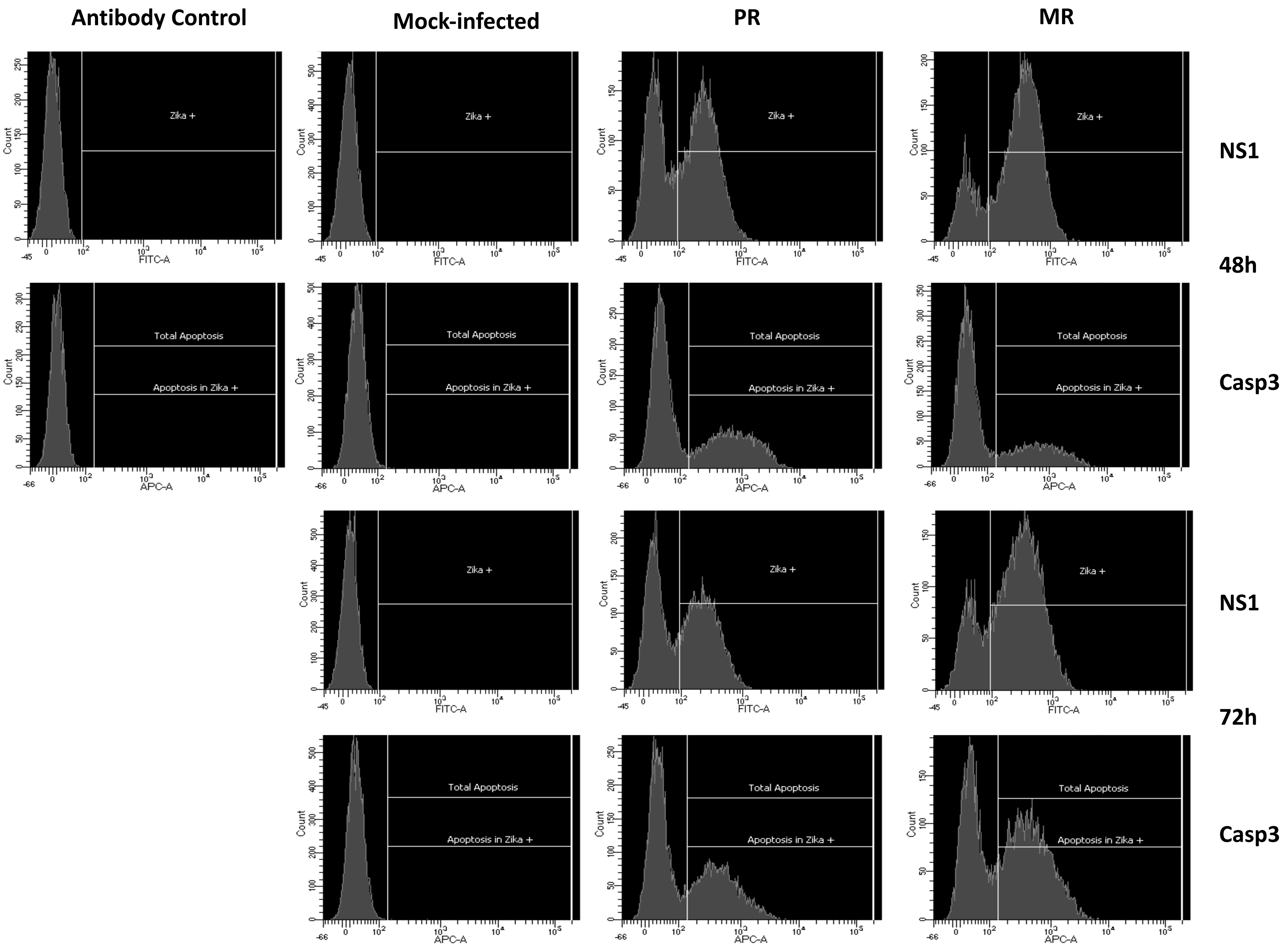

B.

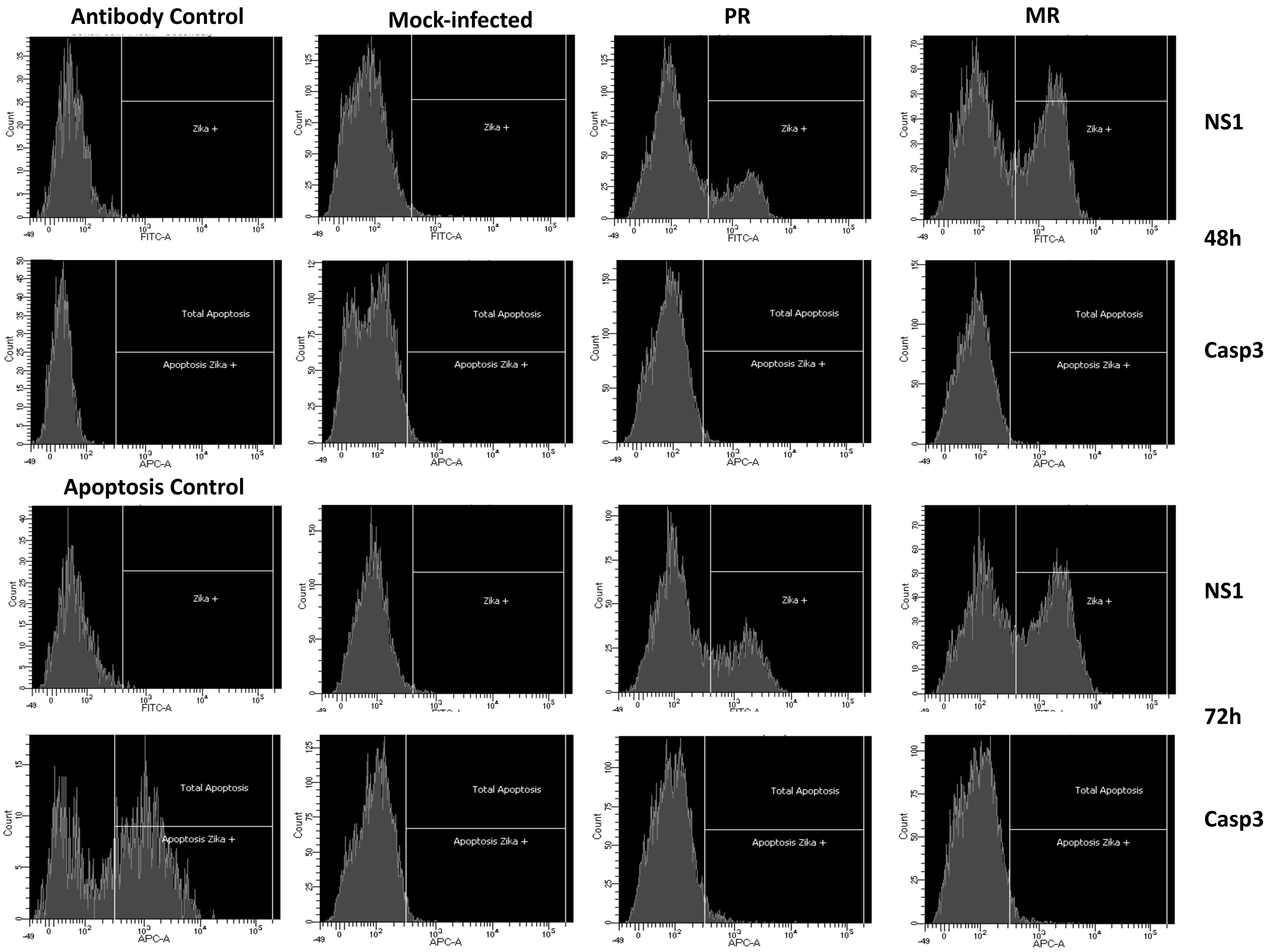

Figure S4. ZIKV infection and apoptosis in A549 and Sertoli cells. (A) A549 and (B) Sertoli cells were infected with ZIKV MR766 (MR) or PRVABC59 (PR) (MOI=5). The cells were harvested at 48 and 72 hours and virus infection levels (intracellular NS1; NS1) and apoptosis (activated caspase 3; Casp3) were determined by FACS. As a positive control to induce apoptosis (Apoptosis control), Sertoli cells were treated for 16 hours with anti-Fas antibody (250ng/ml), PI3K inhibitor (LY294002; 50μM) and cycloheximide (1μg/ml). Representative histogram images are shown. N=3.

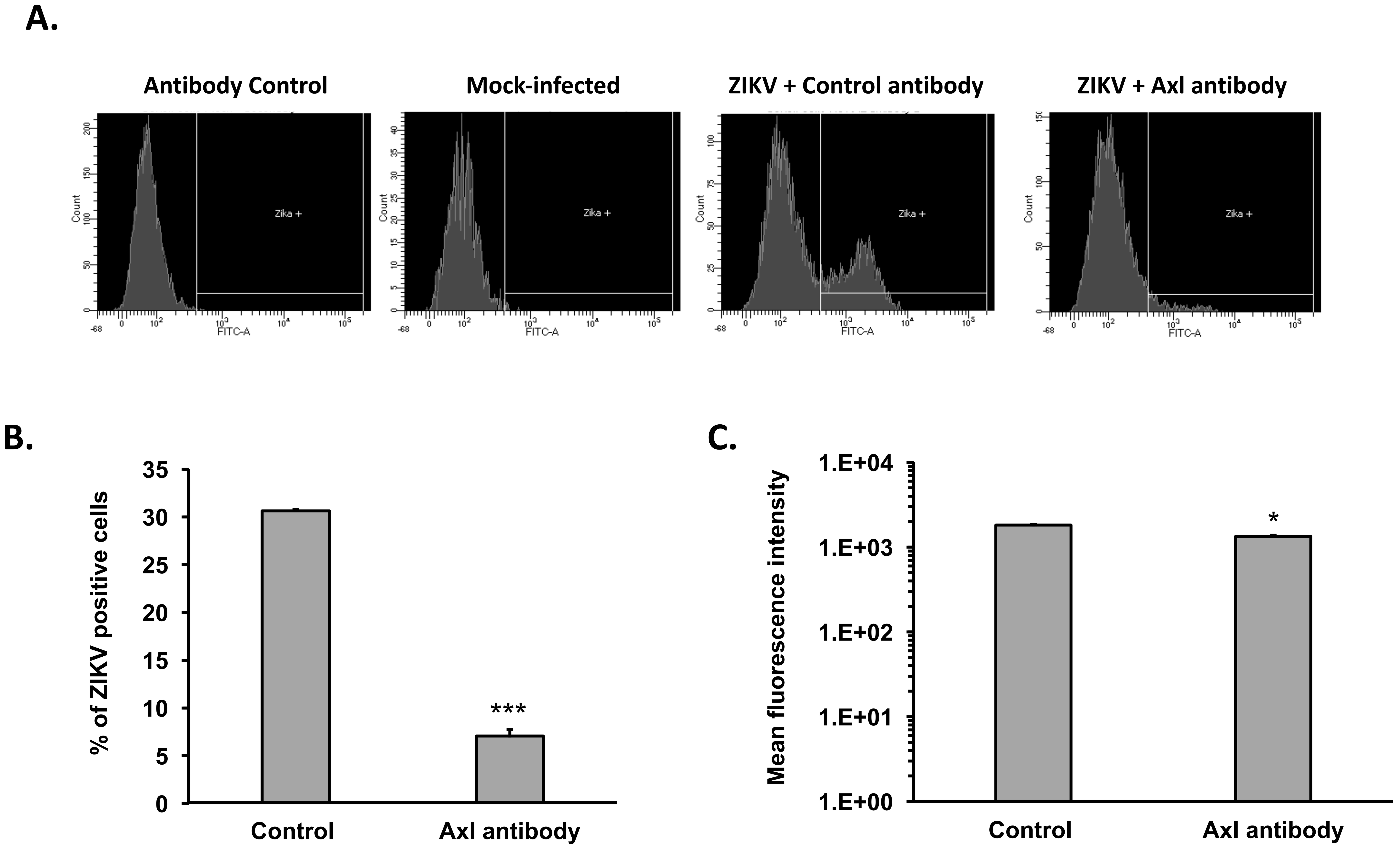

Figure S5. Axl is important for ZIKV entry. Sertoli cells were pre-treated for 2 hours with anti-Axl or control antibodies before infection with ZIKV PRVABC59 (PR) (MOI=5). Forty-eight hours later, cells were harvested and levels of virus infection were determined by FACS using antibodies to NS1. A. Representative histogram images from FACS scans are shown. B. The percentages of ZIKV positive cells and (C) mean NS1 signal intensities are depicted as mean and standard error. *P<0.05, ***P<0.001 (Student's T-test) N=3.

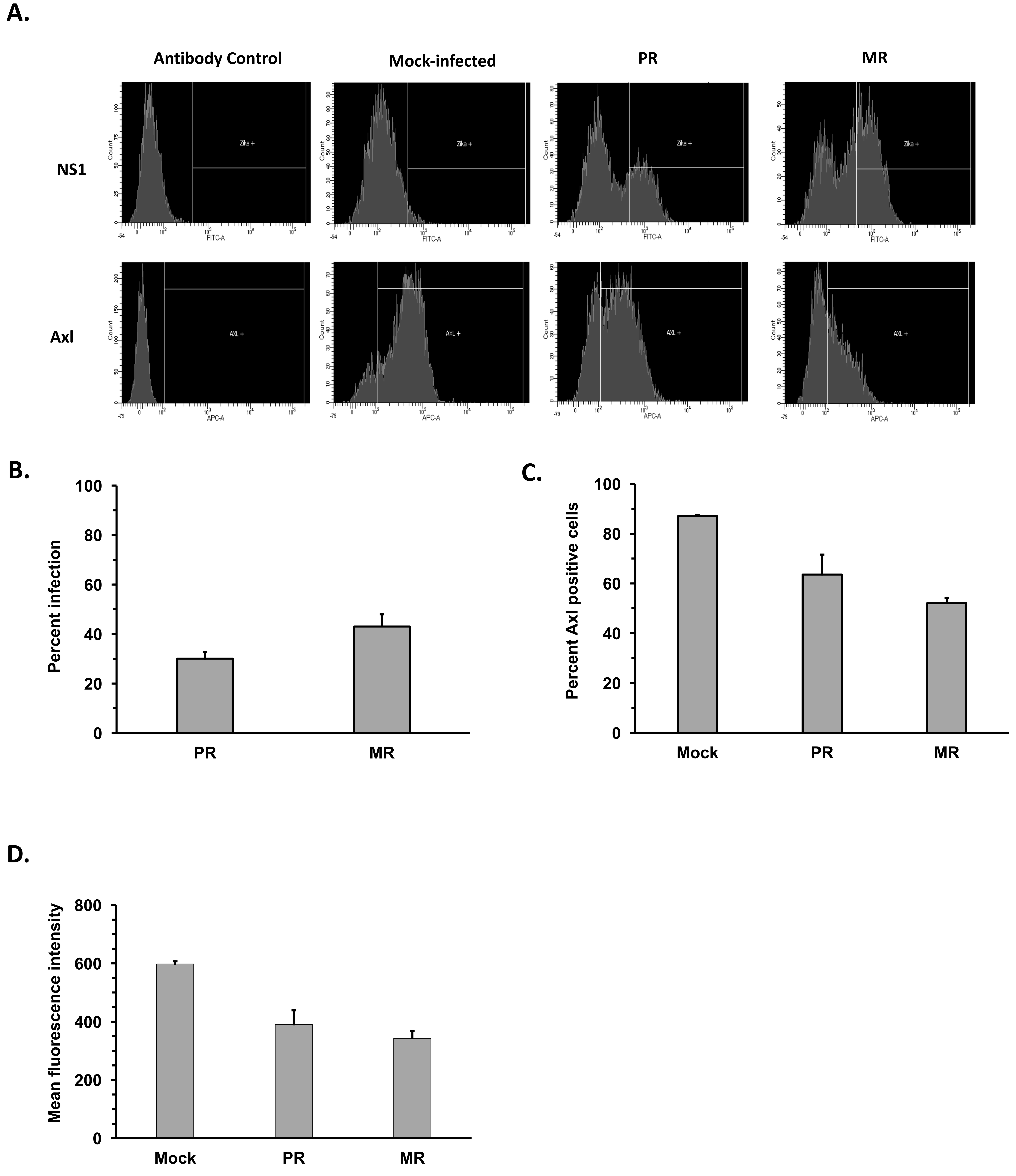

Figure S6. Axl expression is down-regulated in ZIKV-infected cells. Sertoli cells were either mock-treated or infected with ZIKV PRVABC59 (PR) or MR766 (MR) (MOI=5) for 48 hours. Cells were harvested and virus infected cells and Axl expression were assessed by FACS using antibodies to NS1 and Axl respectively. A. Representative histogram images are shown. The percentages of ZIKV-positive (B) and Axl positive cells (C) as well as mean Axl signal intensity (D) are shown as mean and standard error. N=3.

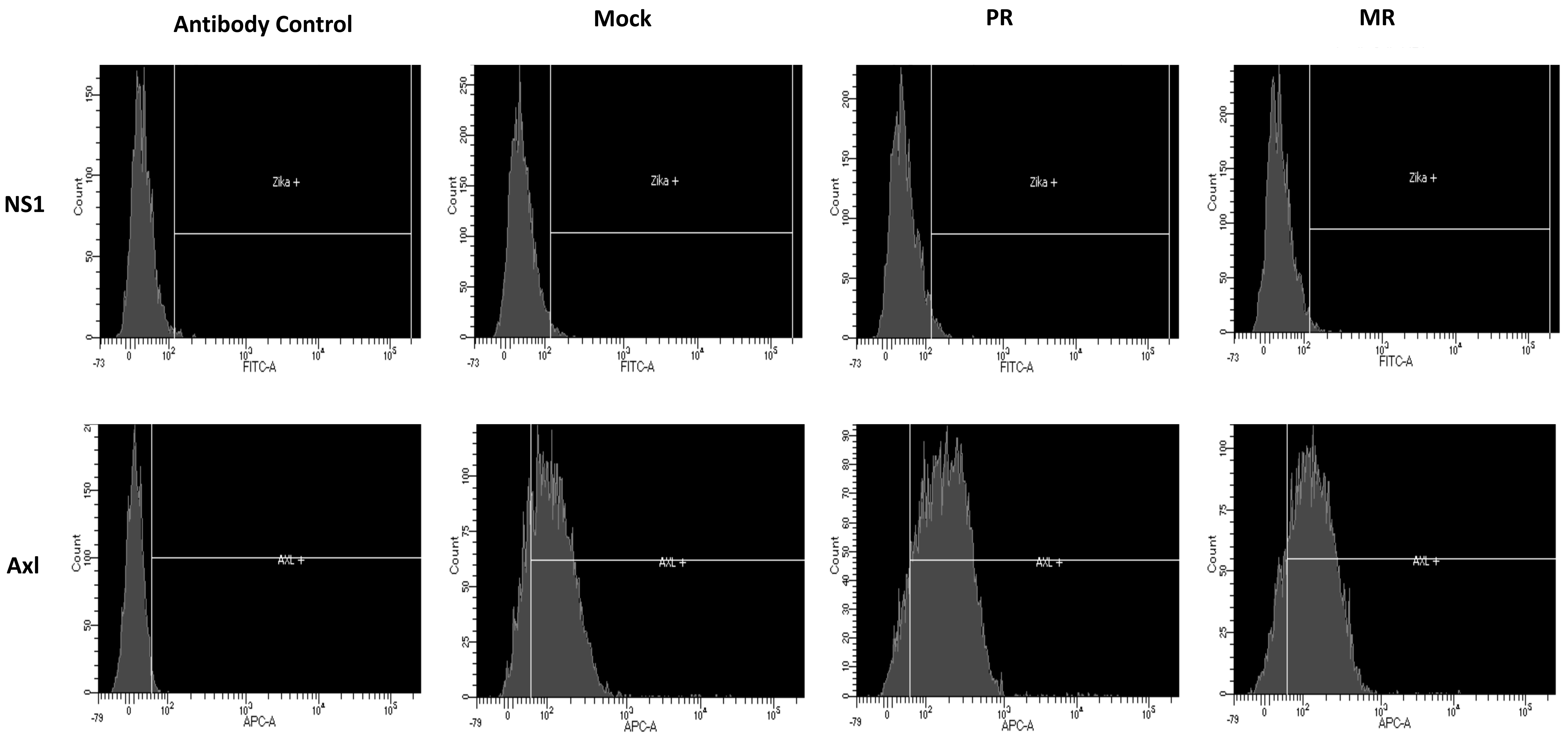

Figure S7. ZIKV infection and Axl expression in Leydig cells. Leydig cells were infected with ZIKV PRVABC59 (PR) or MR766 (MR) (MOI=5) for 72 hours. Cells were harvested and virus-infected cells and Axl expression were assessed by FACS using antibodies to NS1 and Axl respectively. The percentage of Axl(+) cells were 77.77 ± 0.5 (mean and standard error). Representative histogram images are shown. N=3.

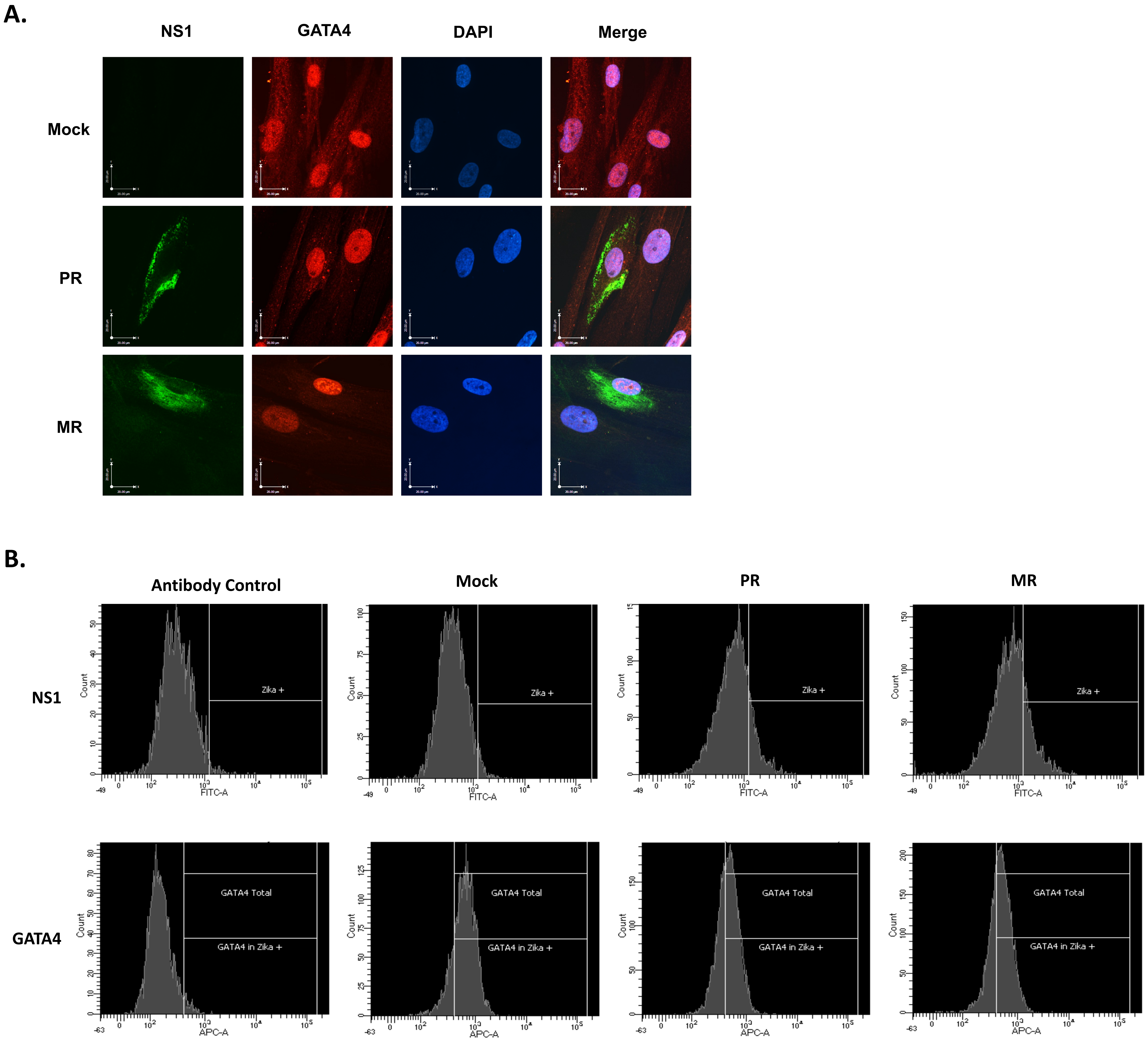

Figure S8. ZIKV persistent infection of Sertoli cells. Sertoli cells were infected with ZIKV PRVABC59 (PR) or MR766 (MR) (MOI=0.5). Forty-two days post-infection cells were harvested and levels of virus infection and GATA4 expression was determined by FACS using antibodies to NS1 and GATA4 respectively. Representative images and histogram images are shown. N=3.

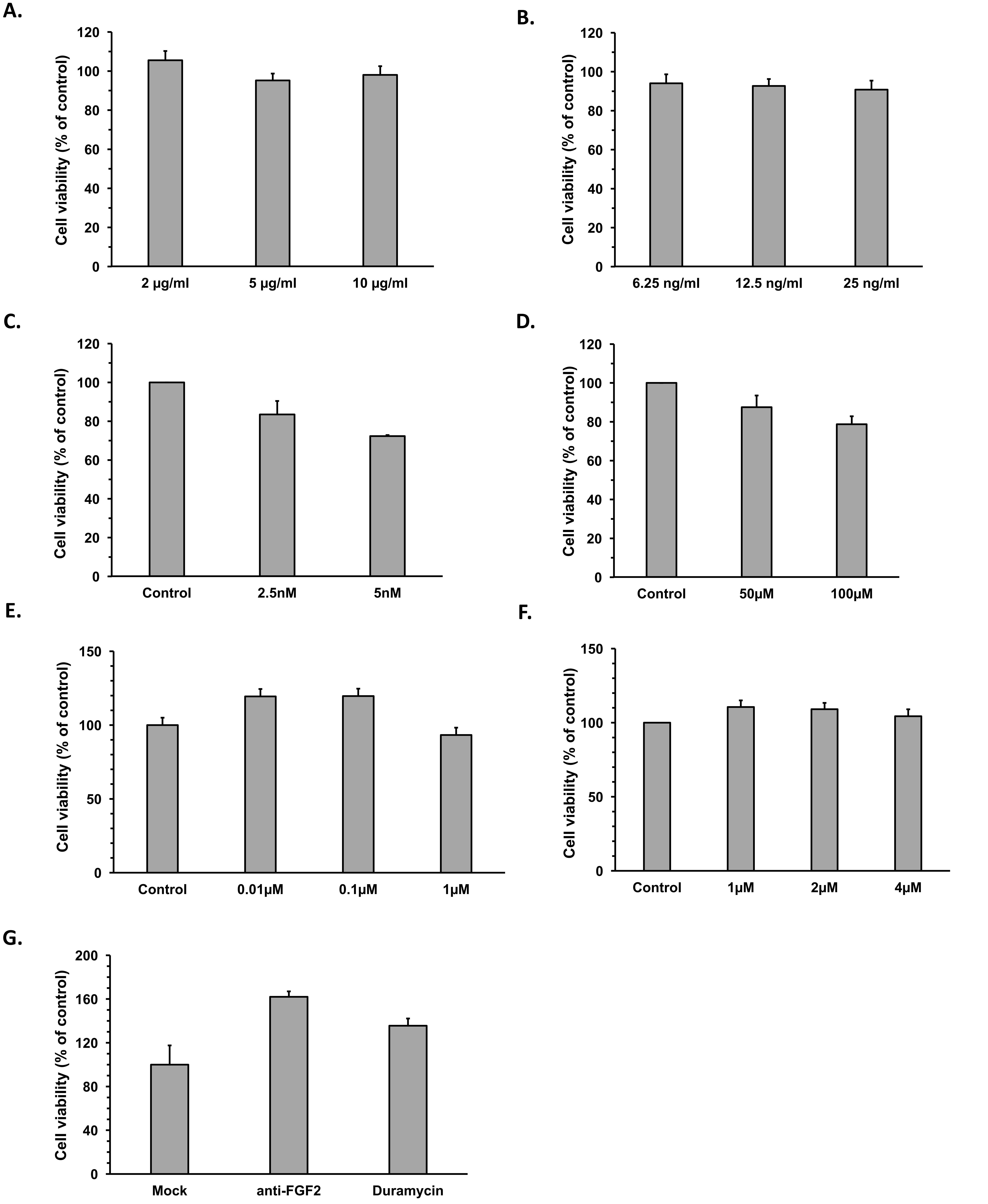

Figure S9. Sertoli cell viability during treatment with FGF2, anti-human FGF2 antibody, FGFR inhibitor BGJ398 and azithromycin. A-D. Sertoli cells were treated for 64 hours with indicated concentrations of (A) anti-human FGF2 antibody, (B) FGF2, (C) FGFR inhibitor BGJ398 or (D) Azithromycin, and for 50 hours with indicated concentrations of (E) Duramycin or (F) R428. **G.** Six-week old mock-infected cell cultures were treated with 0.1µM Duramycin or 10µg/ml anti-human FGF2 antibody for 7-days. Cells were harvested and viabilities were determined using a Cell TiterGlo (Promega) kit. All values are expressed as mean ± standard error. N=3

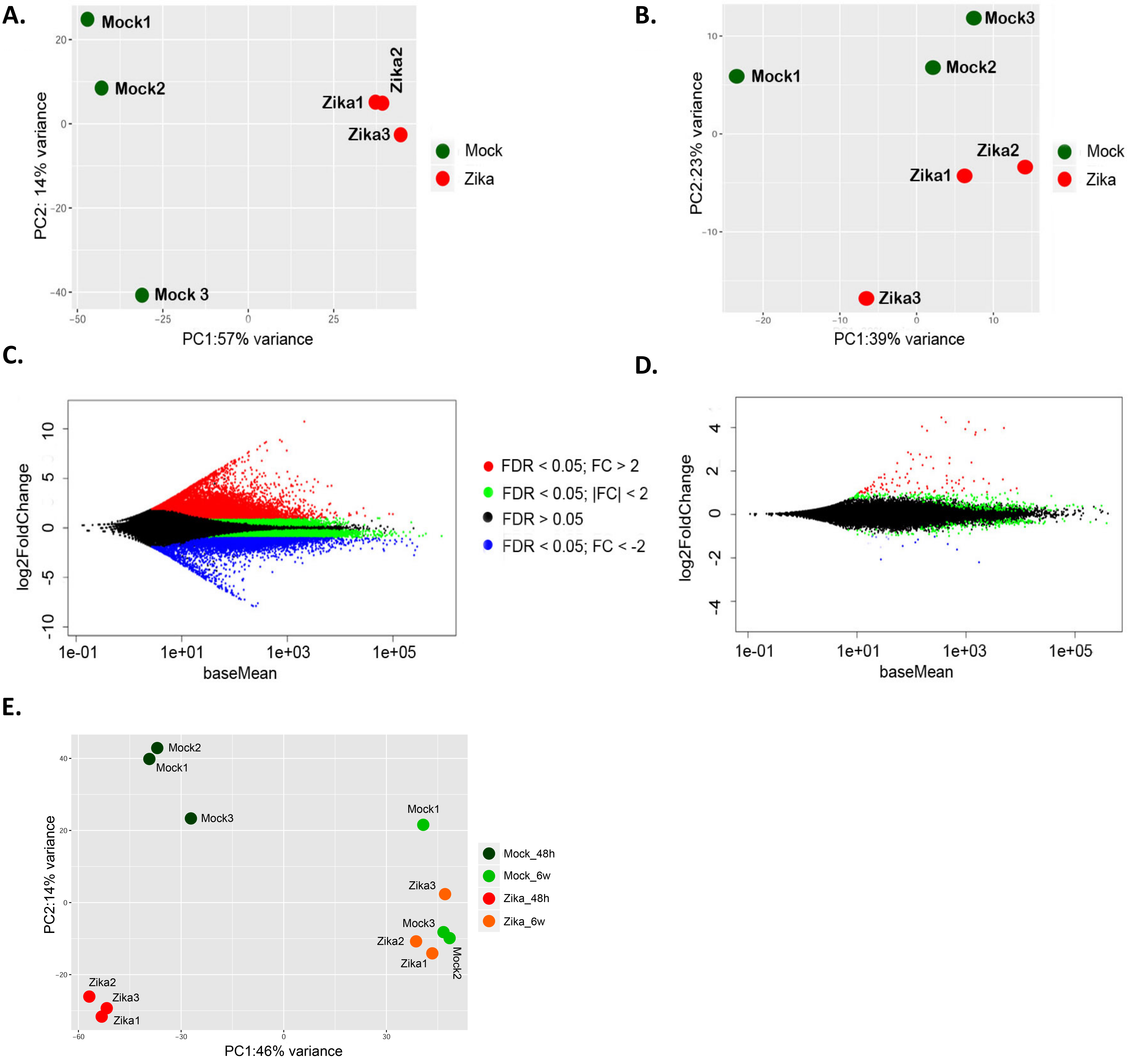

Figure S10. Differential expression analysis reveals massive transcript deregulation in Sertoli cells. Principal component analysis of transcript levels in mock or ZIKV PRVABC59 (PR)-infected cells at 48-hours (A) and 6-weeks post-infection (B) using Euclidian distances. **C-D**. MA plot depicting the log fold-change (Y axis) and the Base Mean (expression level) of transcripts in mock of ZIKV-infected cells at 48-hours (C) and 6-weeks post-infection (D). Red dots represent transcripts with a False discovery rate (FDR) < 0.05 and a Fold change (FC) > 2. Green dots represent transcripts with a FDR < 0.05 and a FC <= 2. Black dots represent transcripts with a FDR > 0.05. Blue dots represent transcripts with a FDR < 0.05 and FC < - 2. E. Differences in the transcriptome profiles at 48-hours and 6-weeks post-infection**.** Principal component analysis of transcript levels in mock or ZIKV Asian strain, PRVABC59 (PR) -infected cells at 48-hours and 6-weeks post-infection using Euclidian distances.

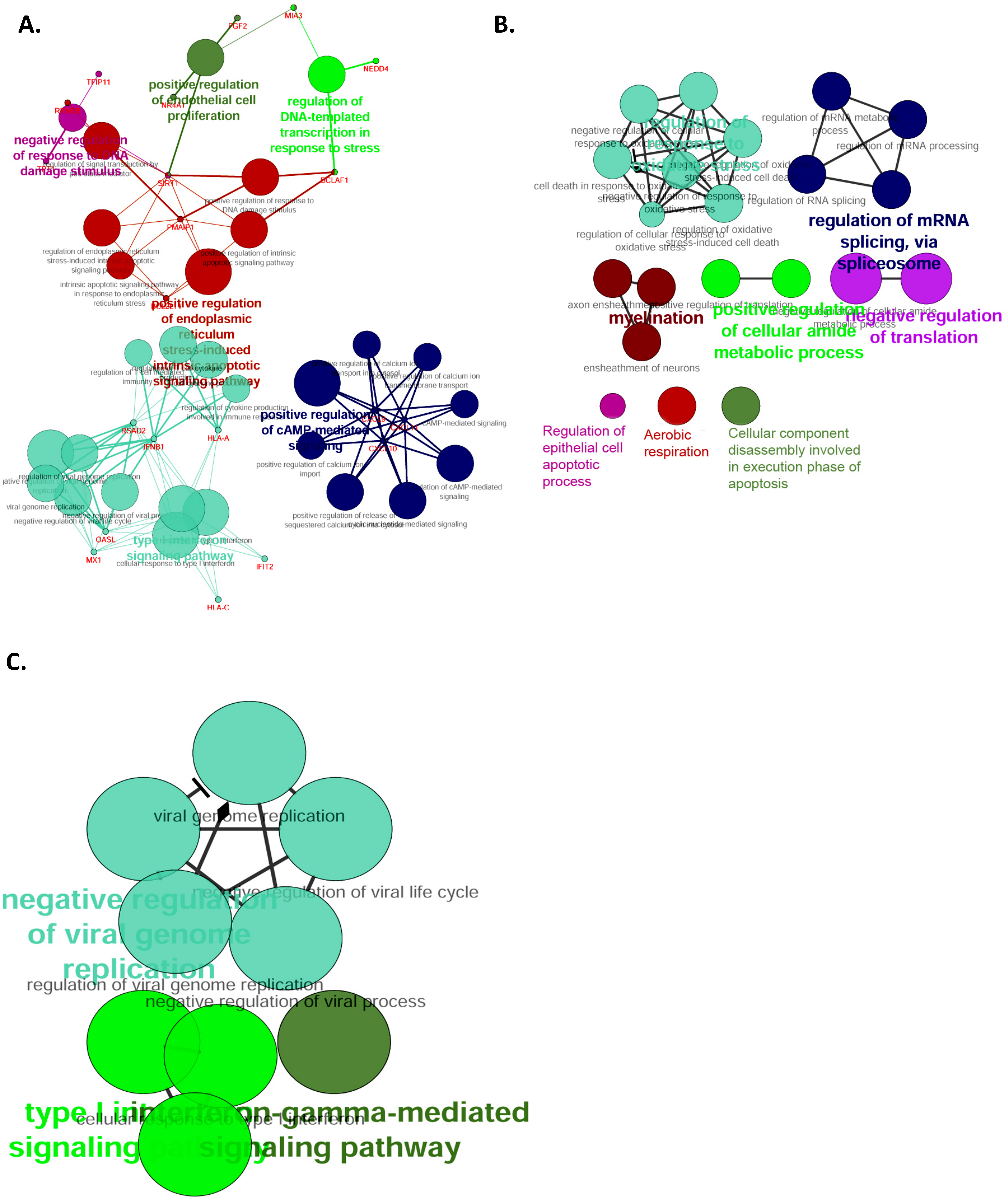

Figure S11. Gene ontology analysis reveals induction of stress responses during acute infection with ZIKV and induction of antiviral defenses during both acute and persistent infection A. Gene ontology results of the 65 top upregulated transcripts (FC > 200) at 48-hours post-infection. **B**. Gene ontology results of the 119 top downregulated transcripts (FC > 100) at 48-hours post-infection. **C**. Gene ontology results of all transcripts found upregulated at 6-weeks post-infection (n = 64). Analysis was conducted with the clueGO PlugIn of Cytoscape. The size of spheres is inversely proportional to the *P*-value of overrepresented terms.

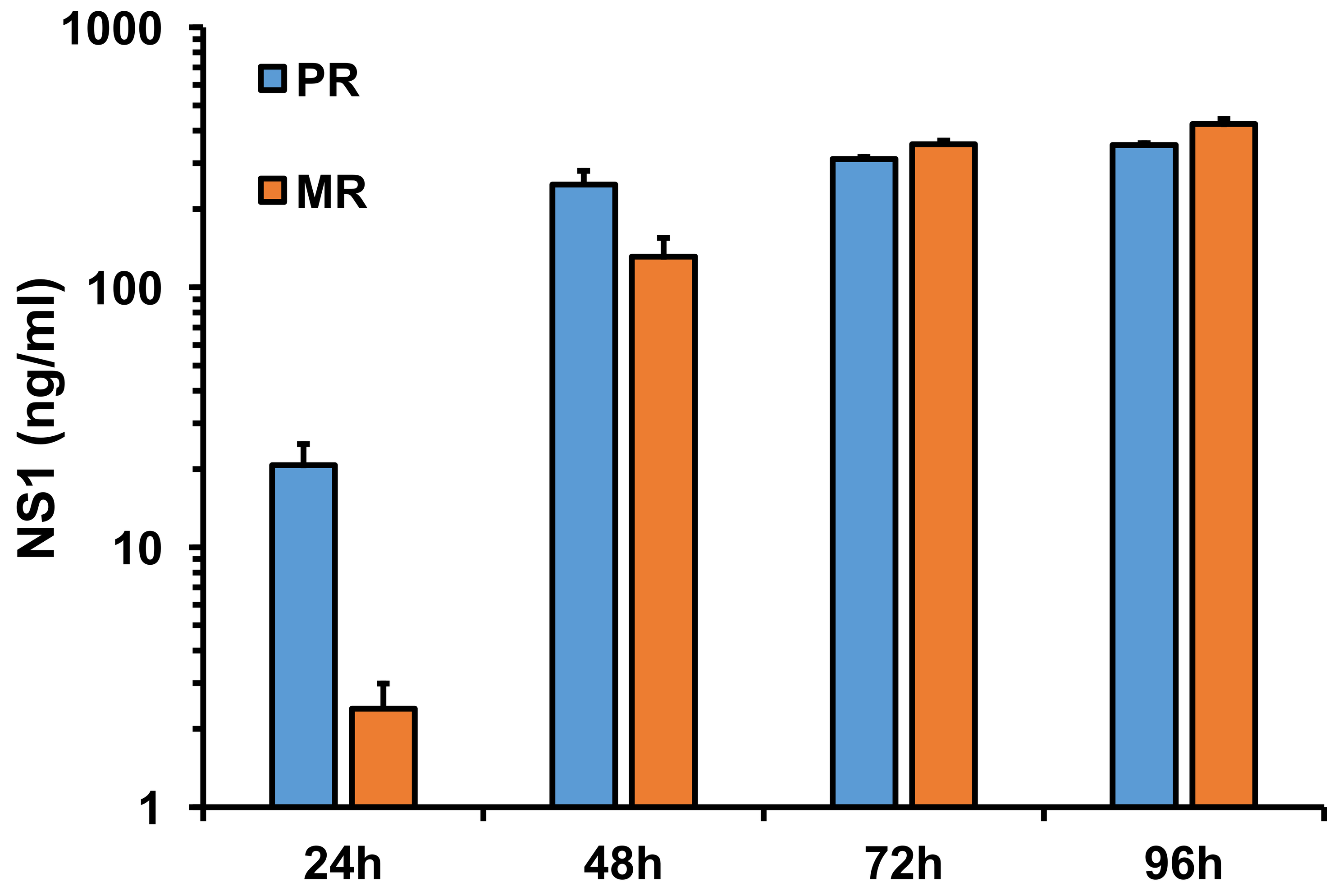

Figure S12. NS1 secretion from ZIKV infected Sertoli cells. Sertoli cells were infected with ZIKV MR766 (MR) or PRVABC59 (PR) (MOI=5) for 24, 48, 72 and 96 hours. At each time point, supernatants were harvested and levels of secreted NS1 were determined by ELISA. N=3.
